# Therapeutic potential of highly functional codon-optimized microutrophin for muscle-specific expression

**DOI:** 10.1038/s41598-022-04892-x

**Published:** 2022-01-17

**Authors:** Anna V. Starikova, Victoria V. Skopenkova, Anna V. Polikarpova, Denis A. Reshetov, Svetlana G. Vassilieva, Oleg A. Velyaev, Anna A. Shmidt, Irina M. Savchenko, Vladislav O. Soldatov, Tatiana V. Egorova, Maryana V. Bardina

**Affiliations:** 1 Laboratory of Modeling and Gene Therapy of Hereditary Diseases, Institute of Gene Biology, Russian Academy of Sciences, Moscow, Russia 119334; 2 Center for Precision Genome Editing and Genetic Technologies for Biomedicine, Institute of Gene Biology, Russian Academy of Sciences, Moscow, Russia 119334; 3 Research Centre for Genetic Medicine, Moscow, Russia 117292; 4 Department of Pharmacology and Clinical Pharmacology, Belgorod State National Research University, Belgorod, Russia 308007; 5 Institute of Gene Biology, Core Facility Centre, Russian Academy of Sciences, Moscow, Russia 119334; 6 Marlin Biotech LLC, Sochi, Russia 354340

**Keywords:** Drug safety, Gene therapy, Neuromuscular disease, Genetic vectors

## Abstract

High expectations have been set on gene therapy with an AAV-delivered shortened version of dystrophin (µDys) for Duchenne muscular dystrophy (DMD), with several drug candidates currently undergoing clinical trials. Safety concerns with this therapeutic approach include the immune response to introduced dystrophin antigens observed in some DMD patients. Recent reports highlighted microutrophin (µUtrn) as a less immunogenic functional dystrophin substitute for gene therapy. In the current study, we created a human codon-optimized µUtrn which was subjected to side-by-side characterization with previously reported mouse and human µUtrn sequences after rAAV9 intramuscular injections in *mdx* mice. Long-term studies with systemic delivery of rAAV9-µUtrn demonstrated robust transgene expression in muscles, with localization to the sarcolemma, functional improvement of muscle performance, decreased creatine kinase levels, and lower immunogenicity as compared to µDys. An extensive toxicity study in wild-type rats did not reveal adverse changes associated with high-dose rAAV9 administration and human codon-optimized µUtrn overexpression. Furthermore, we verified that muscle-specific promoters MHCK7 and SPc5-12 drive a sufficient level of rAAV9-µUtrn expression to ameliorate the dystrophic phenotype in *mdx* mice. Our results provide ground for taking human codon-optimized µUtrn combined with muscle-specific promoters into clinical development as safe and efficient gene therapy for DMD.

## Introduction

Duchenne muscular dystrophy (DMD) is a severe and progressive muscle-wasting disorder affecting one in 5000 boys^1^. It is caused by mutations in the *DMD* gene leading to the loss of dystrophin, a large structural protein located beside the sarcolemma. Gene replacement therapy involving the systemic delivery of a shortened version of dystrophin (microdystrophin, µDys) through recombinant adeno-associated vectors (rAAV) represents a potential approach for DMD treatment^2^. Over 20 years of intensive research has allowed for the development of four rAAV-µDys vectors, which reached clinical trials (NCT03375164, NCT03362502, NCT03368742, and GNT0004). One of the encountered challenges was the generation of immune responses against epitopes in µDys, which may have contributed to a failure to detect dystrophin expression^3^. In light of µDys immunogenicity, utrophin has emerged as a promising target for innovative DMD therapy.

The utrophin protein is a functional paralog of dystrophin with 73% amino acid sequence identity^4^. Full-length utrophin is highly expressed throughout the sarcolemma in fetal muscle. The level of utrophin at the muscle membrane declines during late embryonic stages and becomes restricted to the myotendinous and neuromuscular junctions as well as blood vessels in normal adult muscle^5^. Thereafter, its functions within the sarcolemma are performed by dystrophin, which connects the contractile apparatus and extracellular matrix through the dystrophin-associated glycoprotein complex (DAGC). Similar structure and overlapping localization allow the use utrophin as a highly functional dystrophin substitute^6,7^. Thus, the upregulation of endogenous utrophin via small molecules^8–10^ artificial transcription factors^11,12^, and biglican therapy has been explored for DMD treatment^13,14^. rAAV-based therapies with a miniaturized form of utrophin represent another promising therapeutic approach for DMD.

The limited capacity of AAV vectors necessitates an effective expression cassette with a reduced length of the delivered gene. In 2003, Cerletti and colleagues^15^ performed adenovirus-mediated mini-utrophin gene transfer in Golden Retriever dogs with canine muscular dystrophy. Mini-Utrophin was a synthetic construct (AX107972), with N- and C-terminal regions derived from murine and human cDNA, respectively. Treatment reduced fibrosis and upregulated the expression of dystrophin-associated glycoproteins in affected muscle. In 2008, the Chamberlain lab designed a murine codon-optimized µUtrn ΔR4–R21/ΔCT (EU293093.1), which was identical to a previously reported truncated variant of dystrophin^16,17^. rAAV6-µUtrn improved muscle histopathology and prolonged the lifespan of *mdx:utrn*^*−/−*^ mice. In 2009, Sonnemann et al.^18^ showed that repeated systemic administration of ΔR4-21 µUtrn-TAT protein could attenuate the phenotype of *mdx* mice. Kennedy et al.^19^ later confirmed that AAV9-µUtrn (ΔR4–R21/ΔCT)^17^ could indeed alleviate skeletal and cardiac muscle pathology in D2/*mdx* mice. Recently, Song et al.^20^ carried out an extensive preclinical study with codon-optimized µUtrn delivered to small and large animals via an rAAV9 vector, demonstrating the lower immunogenicity of µUtrn compared to µDys. Banks et al.^21^ reported that rAAV6-CK8e-µUtrn improved dystrophic pathophysiology, with fiber-type expression preferences.

Further, many studies of microdystrophin vectors have demonstrated that codon optimization can significantly increase transgene protein levels^22,23^. Muscle-specific promoters can provide a high, robust expression of the transgene in skeletal muscle, diaphragm, and heart, with limited activity in non-target tissues^24^. While such promoters are used in all clinical trials for microdystrophin delivery^2^, few studies have reported muscle-specific µUtrn expression^20,21^.

In the present study, we sought to further explore the safety and efficacy of AAV-delivered µUtrn constructs. Based on mouse µUtrn configuration^17^, we designed a new human codon-optimized transgene and characterized it in the context of rAAV9-based administration in rodent models. *Mdx* mice were used to assess the efficacy of human codon-optimized µUtrn in alleviating the dystrophic phenotype, and transgene immunogenicity was evaluated relative to that of a microdystrophin-coding construct. Extensive toxicity studies were performed in *mdx* mice and Wistar rats following systemic delivery. Finally, we provide data on the expression and functional activity of codon-optimized µUtrn driven by muscle-specific promoters.

## Results

### Intramuscular administration of rAAV9-μUtrn leads to robust expression of µUtrns and muscle function improvement in ***mdx*** mice

**Figure 1:**
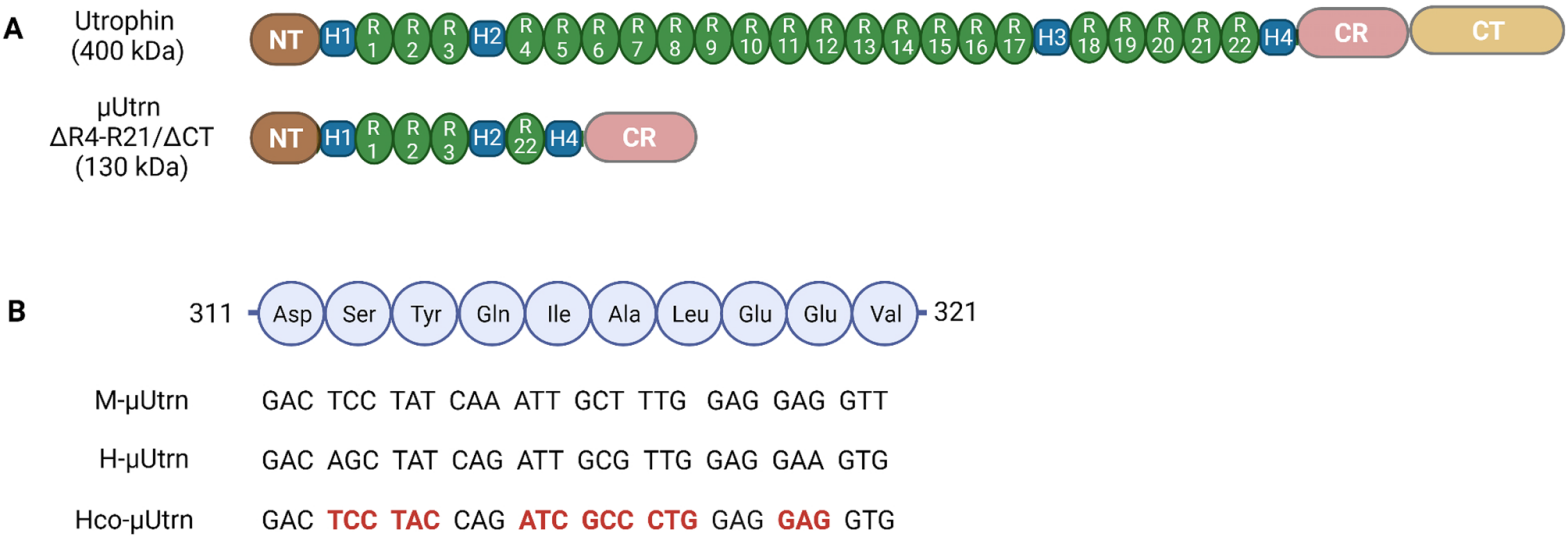
Design of µUtrn-coding sequences. (**A**) Domain structure of full-length utrophin and µUtrn proteins. Domain configuration of µUtrn ∆R4-R21/ΔCT closely resembles microdystrophin R4-R23/ΔCT and consists of an N-terminal actin-binding domain, hinge 1, spectrin-like repeats 1–3, hinge 2, spectrin-like repeat 22, hinge 4, and a cysteine-rich (CR) domain. (**B**) Alignment of the µUtrn coding sequences used in this study. Fragments of spectrin-like repeat 1 coding sequences are shown for mouse (*M-*µ*Utrn*), human (*H-*µ*Utrn*), and codon-optimized human (*Hco-*µ*Utrn*) µUtrns. Codons optimized for expression in muscle are highlighted in bold red.

Previous studies provided compelling evidence that murine and human µUtrns ΔR4-R21/ΔCT (Fig. 1A) can functionally compensate for the lack of dystrophin in mouse models of DMD^17,19–21^. Building on their findings, we designed a human version of µUtrn ΔR4-R21/ΔCT and customized it through codon optimization in order to enhance expression in striated muscles and heart, as described in *Materials and Methods* (Fig. 1B). The human µUtrn sequence had 37% optimal codons, and this index reached 100% after optimization (Supplementary Fig. S1). For direct comparison, the murine, human, and codon-optimized human µ*Utrn* cDNAs (designated in this study as *M-*µ*Utrn*, *H-*µ*Utrn*, and *Hco-*µ*Utrn*, respectively) were cloned into the AAV vector expression cassette under a cytomegalovirus (CMV) promoter. As a control, a similar AAV construct with the cDNA of microdystrophin ΔR4-R23/ΔCT^16^ was created. To produce rAAV viruses encoding µUtrns (rAAV-CMV-µUtrn), we used AAV serotype 9, which is currently being tested in clinical trials for microdystrophin delivery (NCT003362502, NCT03368742).

**Figure 2:**
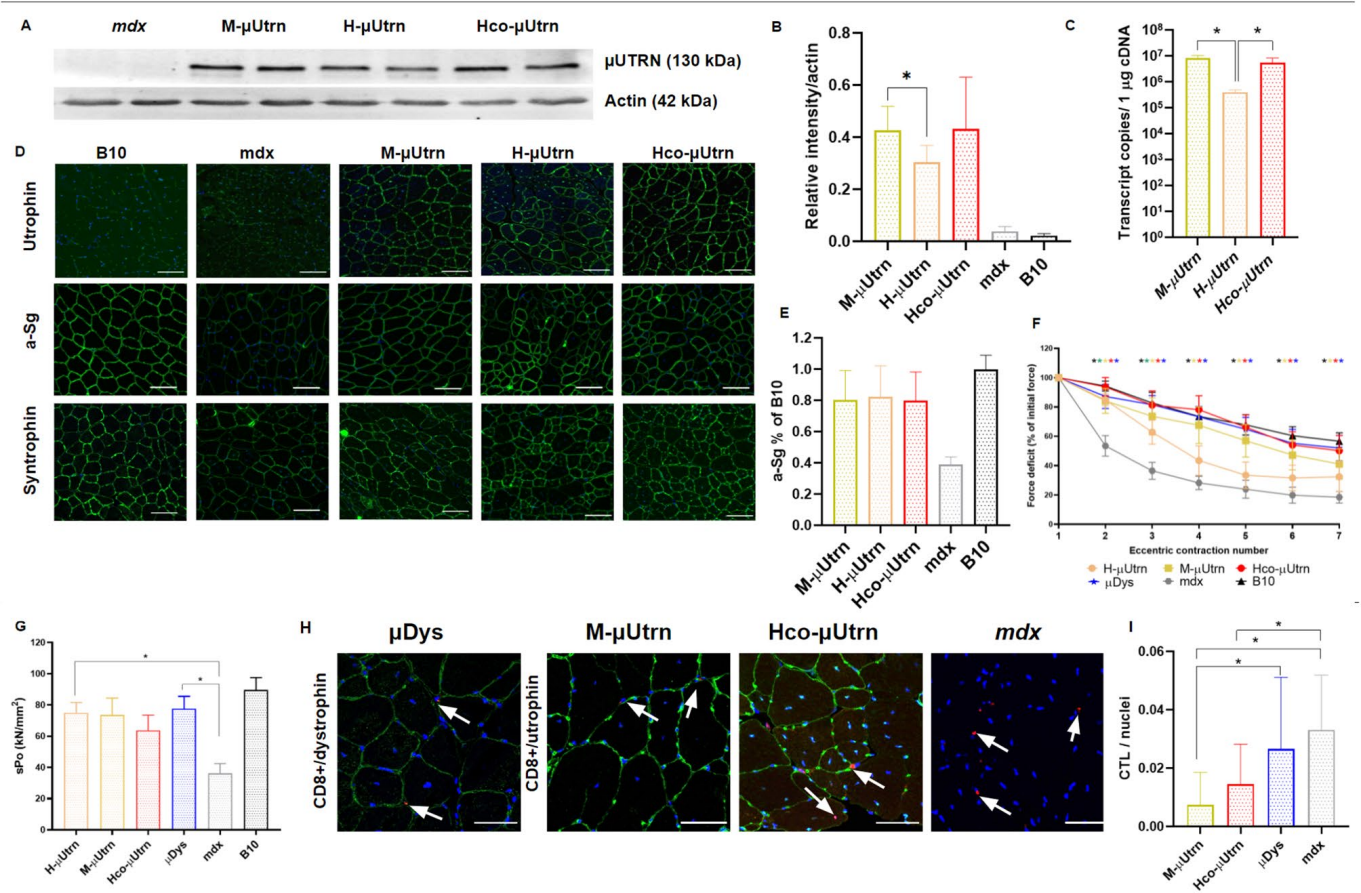
Intramuscular administration of rAAV9-µUtrn at dose 2 × 10^12^ vg/TA muscle leads to robust transgene expression and improves the contractile function of the TA muscle in *mdx* mice after 2 weeks post injection. (**A**) Western blot analysis of recombinant μUtrn expression in tibialis anterior (TA) muscles, with α-actin as the loading control. Unprocessed full-length blots are presented in Supplementary Figure S10. (**B**) Quantitation of µUtrn expression determined via western blot. Data are presented as mean ± SD, * P ≤ 0.05, n = 4/group. (**C**) Analysis of µUtrn transgene expression via RT-qPCR. (**D**) Representative images of native and recombinant utrophin as well as α-sarcoglycan (a-Sg) and α1-syntrophin immunofluorescence in TA muscle cross-sections. Nuclei are counterstained with Hoechst 33,342 (blue). Scale bars, 100 µm. (**E**) Analysis of α-sarcoglycan (a-Sg) expression levels in the sarcolemma of rAAV9-µUtrn-treated muscle in comparison to that in untreated *mdx* and B10 mice. Data are presented as the mean ± SD, n = 4–10 sections/group. (**F**) Percentage force drop following 20% eccentric contraction and (**G**) specific force of TA muscles from *mdx* mice administered rAAV9-µUtrn as compared to that in vehicle control mice. Values in (**C, F**), and (**G**) are presented as the mean ± SEM (N = 8 TA muscles for each group), and statistical significance was set at* P* ≤ 0.05. (**H**) Representative images of CD8 + cytotoxic T-lymphocyte immunofluorescence (red dots marked with white arrows) in TA muscle sections. µUtrn and µDys in the sarcolemma were stained in green, while nuclei were stained in blue. Scale bar, 50 μm. (**I**) Quantitation of CD8 + CTLs in TA muscle sections. CTL number normalized per nuclei, reflecting the cross-sectional area. Values are expressed as the mean ± SD, n = 11–55 sections analyzed per group.

To characterize different µ*Utrn* sequences in dystrophic mice, rAAV9-CMV-µUtrn viruses were administered to 7-week-old *mdx* mice via intramuscular injections of 2 × 10^12^ vg into the tibialis anterior (TA) muscle. rAAV9-CMV-µDys viruses were used as a control. The expression, potency, and immunogenicity of µUtrns were assessed after 2 weeks. As expected, the CMV promoter drove robust mRNA expression in the muscle tissue for all three µUtrn-coding vectors (Fig. 2c). *H-*µ*Utrn* transcripts were the least abundant among *M-*µ*Utrn* and *Hco-*µ*Utrn*. Recombinant utrophin production was confirmed via western blotting analysis with antibodies against the N-terminus of the utrophin protein (Fig. 2A). Quantification of the protein bands revealed lower expression levels for H-μUtrn (Fig. 2B), in agreement with mRNA expression. Importantly, codon usage bias in *Hco-*µ*Utrn* did not compromise protein translation within murine muscles. Immunofluorescence analysis with antibodies against utrophin indicated that µUtrn proteins successfully localized to the sarcolemma of muscle cells, where they can perform their function, substituting dystrophin protein in the DAGC of *mdx* mice (Fig. 2D). Immunostaining of treated muscles with antibodies against DAGC proteins α-sarcoglycan and α1-syntrophin indicated that, in comparison with non-treated *mdx* muscles, the levels of both increased considerably (Fig. 2D,E).

Striking improvements of TA contractile properties were observed in rAAV9-µUtrn-treated *mdx* mice, with specific force increasing roughly twofold relative to that in untreated *mdx* mice (Fig. 2G). Similarly to µDys, H-µUtrn exhibited a pronounced effect. All miniaturized proteins (H-µUtrn, M-µUtrn, Hco-µUtrn, µDys) ensured muscle susceptibility to repeated eccentric contraction at levels comparable to those in control B10 mice (Fig. 2F). Force drop dynamics were indistinguishable between Hco-μUtrn-treated, wild-type control, and µDys group muscles, indicative of the high functionality of the recombinant protein.

In order to verify the lower immunogenicity of utrophin, we assessed the presence of CD8 + cytotoxic T-lymphocytes (CTLs) in muscle sections. Lymphocytes were detected around muscle fibers in all analyzed groups (Fig. 2H), which is typical for the dystrophic environment and has been previously investigated by other groups^25^. We observed a decrease in CTL count in M-µUtrn- and Hco-µUtrn-treated muscles compared to untreated *mdx* muscles reflecting lowering of inflammation, while infiltration rate remained unchanged in the µDys-treated group (Fig. 2I). At the same time µDys-treated muscles showed similar functional recovery as µUtrn-treated, indicating successful expression and functionality of all transgenes (Fig. 2F,G). One can speculate that µDys may be targeted by CTLs to a greater extent than µUtrn, counterbalancing reduced dystrophy-associated infiltration as a consequence of treatment. Moreover, sections of Hco-µUtrn-treated muscle exhibited more CTLs than observed for the M-µUtrn group. A possible reason could be that the mice received human codon-optimized µUtrn, which differs from endogenous murine utrophin. The cellular immune response may be triggered against the AAV capsid as well as the transgene product^3,26^. Since we used the rAAV9 vector for microgene delivery in all groups, we assume that the difference in the number of infiltrating T-cells was associated with the different transgenes. Found observations need further investigation at longer time points and in the context of systemic administration, as intramuscular AAV delivery is known to enhance immune responses^27^.

Side-by-side comparison of µUtrn variants following intramuscular injections in *mdx* mice revealed the potential of rAAV9-Hco-μUtrn, which induced the robust expression of functional protein with lower immunogenicity. Taking into account the potential of microutrophins as a gene therapy for DMD, human codon-optimized µUtrn was chosen for the subsequent experiments.

### Systemic delivery of rAAV9-Hco-μUtrn for long-term studies in *mdx* mice

**Figure 3:**
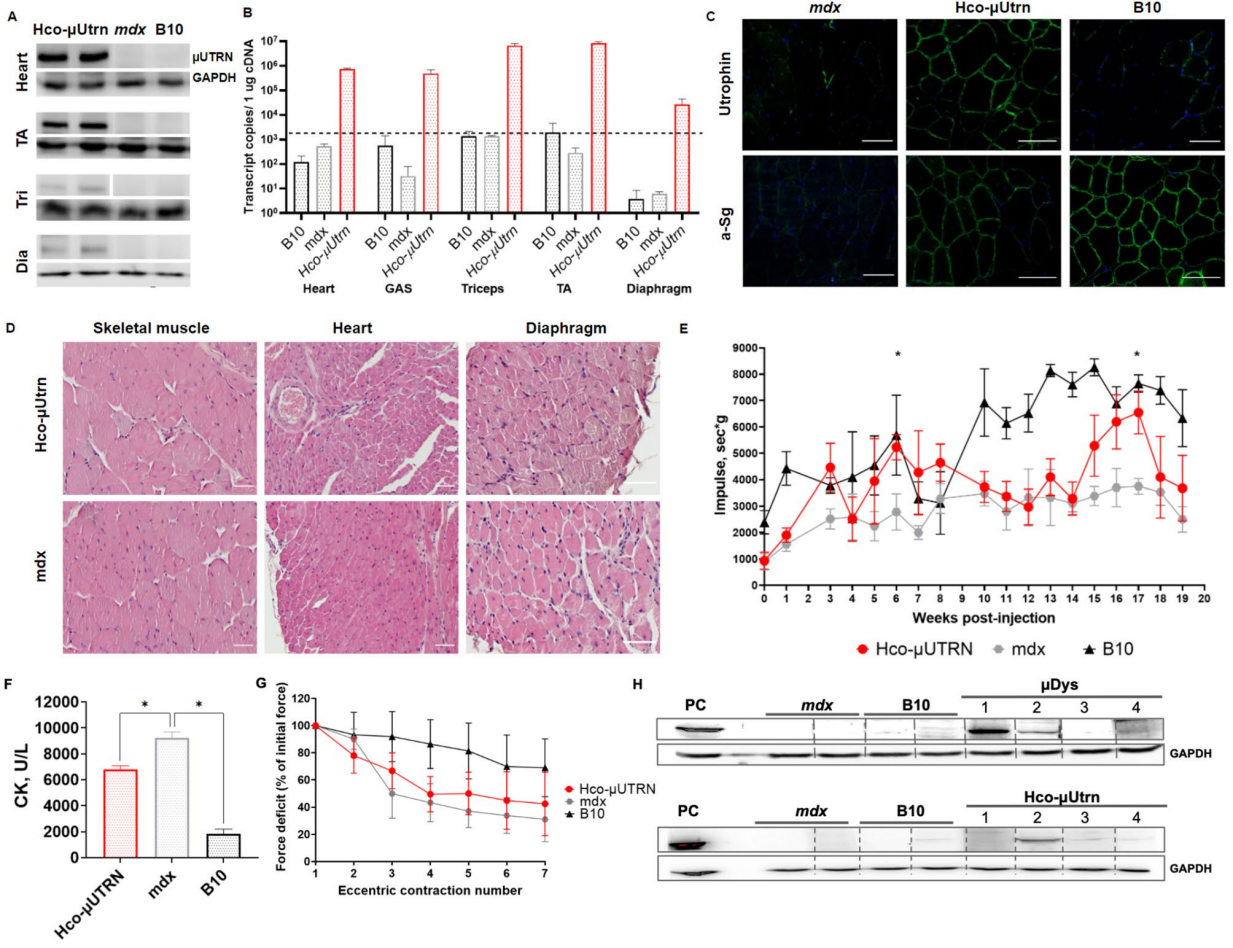
Systemic delivery of rAAV9-Hco-µUtrn at dose 6 × 10^14^ vg kg^−1^ for twenty-week studies in *mdx* mice. (**A**) Western blot analysis of recombinant µUtrn expression in the heart, tibialis anterior (TA), triceps (Tri), and diaphragm (Dia); loading control, GAPDH. Unprocessed full-length blots are presented in Supplementary Figure S10. (**B**) RT-qPCR analysis of Hco-µUtrn expression in the heart, gastrocnemius (GAS), triceps, TA, and diaphragm. The dashed line represents the detection levels of negative controls. (**C**) Representative images of TA muscle cryosection immunofluorescence analysis after rAAV9-Hco-µUtrn administration. Native and recombinant utrophin as well as α-sarcoglycan were colored in green. Nuclei were counterstained with DAPI (blue). Scale bar, 100 µm. (**D**) Representative images of hematoxylin and eosin (H&E)-stained skeletal muscle, heart, and diaphragm (see Fig. S2 for more organs). Scale bar, 100 µm. (**E**) Hanging wire test. The maximum hanging time of three trials during a 300-s wire test protocol normalized to mouse mass. (**F**) Creatine kinase (CK) levels in serum. (**G**) Percentage force drop following 20% eccentric contraction of TA muscles of *mdx* mice administered rAAV9-Hco-µUtrn compared to those of vehicle control mice. (**H**) Western blot analysis of Hco-µUtrn- and µDys-specific antibodies in the sera of treated mice (1:100 dilution). PC—positive control, sample incubated with antibodies against utrophin (Cau22354) and dystrophin (DysB); loading control, GAPDH. Unprocessed full-length blots are presented in Supplementary Figure S10. All values are presented as the mean ± SEM (n = 4 mice), and statistical significance was set at *P* < 0.05.

In order to explore the systemic effects of transgene delivery, 6-week-old *mdx* mice were injected with rAAV9-Hco-µUtrn at a dose 6 × 10^14^ vg kg^−1^. The twenty-week study included functional testing throughout the experiment (the hanging wire test) and multiple terminal examinations such as transgene expression, muscle histopathology, CK levels, force deficit, and humoral immunity assessment. Protein and RNA expression analysis confirmed the successful AAV9-mediated delivery of transgenes to all target organs, including striated muscle, heart, and diaphragm (Fig. 3A,B). Transcript levels in TA muscle were comparable to those detected after direct intramuscular injection. Human codon-optimized µUtrn was efficiently delivered to the sarcolemma, thus restoring the DAGC (Fig. 3C).

Morphological analysis of skeletal muscle, heart, and diaphragm showed myofiber damage and many centronucleated muscle cells, in agreement with the histopathological aspects of the disease. There were no prominent differences between treated and untreated *mdx* mice. Features observed in all mice from both groups included areas of necrosis and regeneration as well as sites of inflammation and fibrosis, which are typical for DMD (Fig. 3D). There were no changes in other organs, except for the thyroid (Supplementary Fig. S2). One mouse exhibited a reduction in follicle number and hypertrophy of follicle epithelium 20 weeks after the injection of human codon-optimized µ*Utrn*. We cannot definitively conclude whether these changes were related to rAAV9-Hco-µUtrn treatment or were individual age-related changes. It was previously shown that the systemic delivery of rAAV-based therapeutics can result in hypertrophy of the thyroid epithelium due to a reduction in thyroid hormone levels^28^.

Long-term functional assessment determined via hanging wire tests showed significant improvements in the group of *mdx* mice treated with rAAV9-Hco-µUtrn at several time points (Fig. 3E, weeks 6 and 17). Unexpectedly, on other weeks, functional improvements in the Hco-µUtrn group were not significant, if any. In addition, at week 8, wild-type animals did not exhibit any difference from model mice due to unknown reasons (Fig. 3E). We suppose that the small number of animals in the experimental groups (n = 4) did not allow to exclude the influence of individual characteristics of animals and that greatly distorts the average value in the presence of outliers. Serum CK levels were markedly reduced to 6000–7000 in treated *mdx* mice, as compared to approximately 9000 U/L in control *mdx* mice (Fig. 3F). The specific force of isolated skeletal muscles from rAAV9-Hco-µUtrn–injected mice was higher than that of untreated *mdx*. Similarly, moderate force drop improvements after repeated eccentric contractions were detected in treated animals, although not significant (Fig. 3G).

To assess the humoral immune response to long-term transgene expression, we detected Hco-μUtrn-specific antibodies in the serum of treated animals. In parallel, we analyzed the sera of animals treated with the same dose of AAV9-µDys. We detected µDys-specific antibodies in sera from three of four AAV9-CMV-µDys-injected mice (Fig. 3H, №1, 2, 4 of the µDys group). Antibody levels varied between samples, as indicated by the intensity of bands. The sera of wild-type (B10) and *mdx* mice treated with DPBS did not contain transgene-specific antibodies. Hco-µUtrn-specific antibodies were identified in sera from two out of four treated mice (Fig. 3H, №2, 3 of the µUtrn group). However, band intensity was notably lower than for µDys-treated animals.

Taken together, the long-term monitoring of *mdx* mice after intravenous injection of rAAV9-Hco-µUtrn demonstrated persistent transgene expression in the skeletal muscle, heart, and diaphragm, with appropriate localization to the sarcolemma. Expression of *Hco-*µ*Utrn* allowed for functional recovery and protected muscle against contraction-induced damage. Further, the histopathological analysis did not reveal any toxicity effect associated with the expression of rAAV9-Hco-µUtrn and vector administration at high doses.

### Intramuscular injection of rAAV9-Hco-µUtrn-FLAG virus led to expression on the sarcolemma of TA muscles and functional improvements in ***mdx*** mice

**Figure 4:**
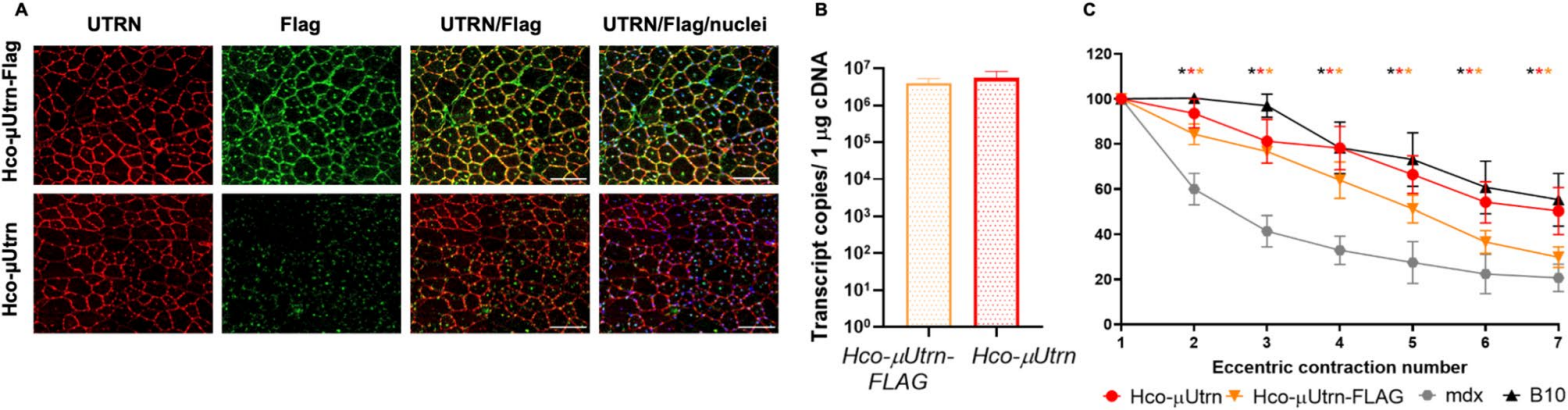
Addition of FLAG epitope to Hco-µUtrn does not interfere with the expression, localization, and function of the recombinant protein after intramuscular administration at dose 2 × 10^12^ vg/TA muscle. (**A**) Representative images of utrophin (red) and FLAG-epitope (green) immunofluorescence in TA muscles of *mdx* mice after 2 weeks post rAAV9-Hco-µUtrn and rAAV9-Hco-µUtrn-FLAG administration. Nuclei were counterstained with Hoechst 33,342 (blue). Scale bar, 100 μm. (**B**) Comparison of µUtrn and µUtrn-FLAG expression level in treated *mdx* TA muscles, as determined via RT-qPCR. Data are presented in transcript copies per 1 µg cDNA. (**C**) Percentage force drop following 20% eccentric contractions of rAAV9-Hco-µUtrn and rAAV9-Hco-µUtrn-FLAG-treated *mdx* TA muscle vs untreated *mdx* and B10 mouse muscle. All values are presented as the mean ± SEM (n = 8), and statistical significance was set at *P* ≤ 0.05.

To distinguish between native utrophin and recombinant µUtrn during immunofluorescence, the FLAG epitope tag was used as a transgene marker. The addition of an N-terminal FLAG tag to the µUtrn sequence has been reported previously^20,21^. In the present study, the Hco-µUtrn protein C-terminus was modified with the DYKDDDDK peptide sequence, allowing for detection of the transgene using antibodies against the utrophin N-terminus and the FLAG epitope. To determine whether the FLAG-tag had a negative impact on Hco-µUtrn expression, localization, and functionality, we injected rAAV9-Hco-µUtrn and rAAV9-Hco-µUtrn-FLAG into the TA muscles of *mdx* mice (2 × 10^12^ vg per TA muscle). Two weeks post-injection, transcript levels and function were analyzed. Hco-µUtrn-FLAG as well as Hco-µUtrn were successfully expressed on the sarcolemma of TA muscle cells (Fig. 4A, UTRN). Antibodies against the FLAG epitope recognized the Hco-µUtrn-FLAG protein (Fig. 4A, FLAG). The expression level was similar for both transgenes (Fig. 4B). Further, both rAAV9-Hco-µUtrn- and rAAV9-Hco-µUtrn-FLAG-treated *mdx* TA muscles exhibited a statistically significant improvement in contractile function when compared to untreated *mdx* mouse muscles (Fig. 4C). Thus, FLAG-tagged Hco-µUtrn can be used for further experiments in *mdx* mice or other animals with endogenous utrophin expression.

### High-dose rAAV9-Hco-µUtrn-FLAG administration does not cause toxicity in rats

**Figure 5:**
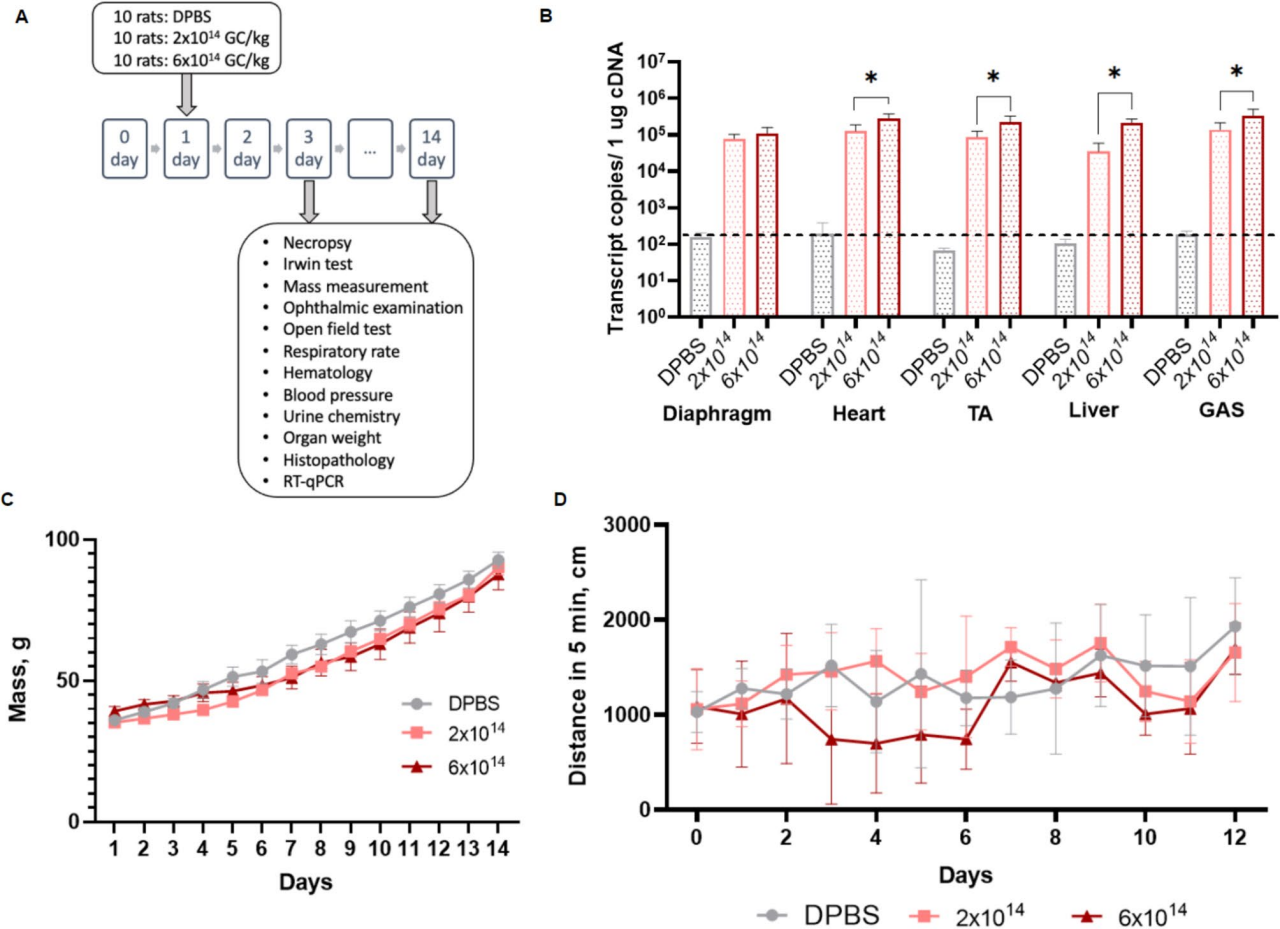
Systemic administration of rAAV9-Hco-µUtrn-FLAG at doses 2 × 10^12^ and 6 × 10^12^ vg kg^−1^ does not cause toxicity in rats after 3 and 14 days post injection. (**A**) Study design. (**B**) Transgene expression levels in the diaphragm, heart, tibialis anterior (TA), liver, and gastrocnemius (GAS) of rats, as determined via RT-qPCR. Values are expressed as transcript copies per 1 µg cDNA. The dashed line represents the detection levels of negative controls. (**C**) Body weight changes in experimental and control animals. (**D**) Distance traveled in 5 min during the open field test. All values are presented as the mean ± SEM (n = 5/time point). Statistical significance was set at *P* ≤ 0.05.

In order to assess the toxicity of human codon-optimized µUtrn delivered via the rAAV9 vector, we administered rAAV9-Hco-µUtrn-FLAG to male rats through intravenous injection. Low (2 × 10^14^ vg kg^−1^) and high (6 × 10^14^ vg kg^−1^) doses were tested. Weaning rats were injected at 3 weeks of age, followed by extensive daily monitoring. Acute and subacute toxicity evaluation was performed on days 3 and 14, respectively (Fig. 5A).

All animals survived the duration of the study, with no significant changes in clinical signs, organ weights, histopathological findings, hematological and coagulation parameters, as well as urine biochemistry. Biochemical blood tests indicated that the levels of ALT, AST, ALP, and GGT did not differ between treated and control rats (Table 1). There was a significant increase in creatinine levels on day 3 in the group receiving a low dose (Table 1), but there was no difference on day 14. The groups did not differ in mass and total activity, as determined via the open field test (Fig. 5D). Daily monitoring of animals confirmed no changes in the condition of the skin, hair, and visible mucous membranes. Ophthalmoscopic examination, ECG recording, urine biochemistry and Irwin tests also confirmed the lack of toxicity associated with rAAV9-Hco-µUtrn-FLAG (Supplementary Fig. S5–S7).

**Table 1:** Results of blood biochemical test in rats on days 3 and 14 after a single intravenous injection of rAAV9-Hco-µUtrn-FLAG.

Day, group	Day 3			Day 14		
	DPBS	2 × 10^14^ vg kg^−1^	6 × 10^14^ vg kg^−1^	DPBS	2 × 10^14^ vg kg^−1^	6 × 10^14^ vg kg^−1^
Albumin, g/L	27.0 ± 4.6	29.4 ± 2.9	25.4 ± 20,0	28.8 ± 2.3	27.8 ± 3.1	26.8 ± 2.2
Total protein, g/L	38.2 ± 5.7	35.6 ± 2.7	40.8 ± 1.8	44.4 ± 4.3	43.9 ± 4.9	39.1 ± 3.1
Creatinine, µmol/L	25.9 ± 2.1	34.2 ± 3.6#	29.6 ± 1.6†	30.2 ± 3.1	29.2 ± 4.7	30.3 ± 4.0
Total cholesterol, mmol/L	2.3 ± 0.3	2.1 ± 0.2	2.1 ± 0.3	1.9 ± 0.1	2.1 ± 0.2	2.0 ± 0.2
Glucose, mmol/L	8.8 ± 0.8	8.3 ± 0.8	8.3 ± 1.0	9.9 ± 3.2	10.6 ± 1.7	8.1 ± 1.8
ALT, IU/L	64.3 ± 9.7	64.6 ± 13.3	57.6 ± 16.5	79.1 ± 12.6	71.1 ± 11.4	80.2 ± 9.7
AST, IU/L	154.9 ± 48.7	227.5 ± 50.6	210.2 ± 50.6	201.0 ± 86.4	184.1 ± 71.7	150.7 ± 26.1
GGT, IU/L	0.3 ± 0.6	0.1 ± 0.1	0.1 ± 0.1	1.0 ± 0.9	0.8 ± 0.9	0.3 ± 0.3
ALP, IU/L	716.1 ± 148.6	633.4 ± 139.4	657.6 ± 166.9	648,0 ± 180.7	677.1 ± 127.5	673.3 ± 176.8
K^+^, mmol/L	5.2 ± 0.6	6.0 ± 1.1	5.9 ± 0.7	5.6 ± 1.6	5.5 ± 1.3	4.5 ± 0.5
Na^+^, mmol/L	142.8 ± 4.4	147.5 ± 7.5	143.0 ± 3.6	143.7 ± 3.9	146.5 ± 10.0	142.5 ± 1.8

All data are presented as mean ± SD.

#*P* = 0.0007 when compared to the control group (DPBS, day 3).

†*P* = 0.0380 when compared to the group treated with a low dose of rAAV9-Hco-µUtrn-FLAG (2 × 10^14^ vg kg^−1^, day 3). In both cases (# and †), statistical analysis was performed via ANOVA and Tukey’s post-hoc test.

At the two-week time point, several organs from the treated animals were subjected to transgene expression analysis. The *Hco-*µ*Utrn* transcript was detected in the diaphragm, heart, TA, gastrocnemius (GAS), as well as the liver, thus confirming successful rAAV9-mediated delivery and efficient expression (Fig. 5B). Dose-dependent expression was evident in all analyzed organs, except for the diaphragm. The high level of transgene transcripts in the liver can be explained by the presence of the constitutive CMV promoter in the transgene construct.

Taken together, our toxicity study confirmed that neither the rAAV9-Hco-µUtrn-FLAG vector itself nor *Hco-*µ*Utrn* expression exhibited toxicity in rats.

### Muscle-specific SPc5-12 and MHCK7 promoters drive µUtrn expression and ensure force improvement after intramuscular injection in ***mdx*** mice

To further reduce immunogenicity, we decided to test an *Hco-*µ*Utrn* construct under muscle-specific promoters. To this end, we replaced the constitutive CMV promoter with MHCK7 and SPc5-12 promoters^29,30^. In contrast to previous reports^20^, including a SPc5-12 promoter in the µUtrn-expressing construct did not lead to the reduction of AAV vector yield (Supplementary Table S8). The modified constructs were administered to 7-week-old *mdx* mice via intramuscular injection at a dose of 2 × 10^12^ vg per TA muscle.

**Figure 6:**
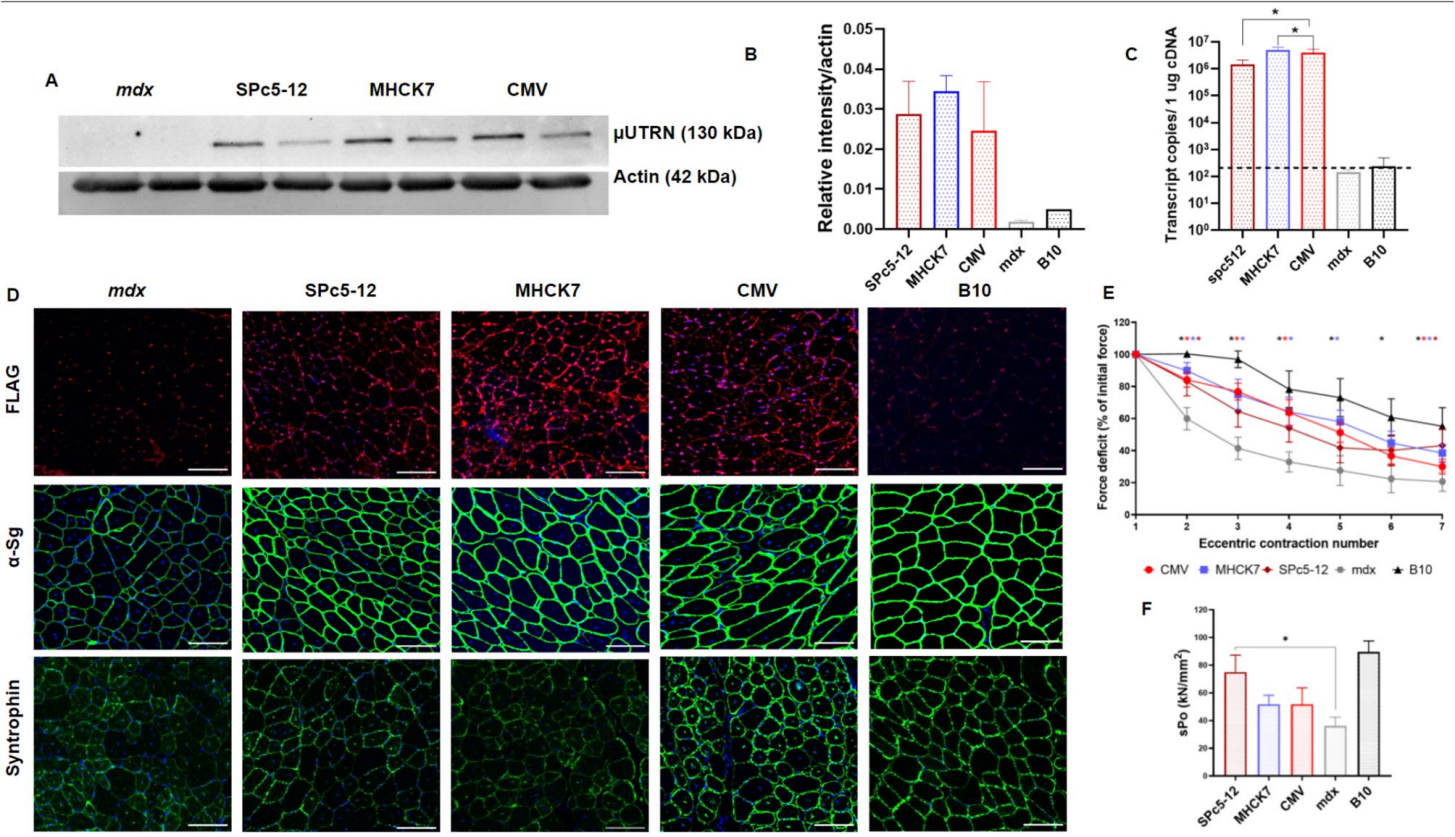
Muscle-specific SPc5-12 and MHCK7 promoters drive µUtrn expression and ensure force improvement after intramuscular injection at dose 2 × 10^12^ vg/TA in *mdx* mice. (**A**) Western blot analysis of recombinant µUtrn expression in TA muscles after 2 weeks post injection, with α-actin as the loading control. Unprocessed full-length blots are presented in Supplementary Figure S10. (**B**) Hco-μUtrn protein levels in rAAV9-Promoter-Hco-µUtrn-treated *mdx* TA muscles determined via western blot and normalized to α-actin levels. (**C**) Analysis of µUtrn transgene expression via RT-qPCR. Values are expressed as transcript copies per 1 µg cDNA. The dashed line represents the detection levels of negative controls. (**D**) Representative images of µUtrn-FLAG (FLAG), α-sarcoglycan (a-Sg), and α1-syntrophin immunofluorescence in TA muscle cross-sections. Nuclei were counterstained with Hoechst 33,342 (blue). Scale bars, 100 μm. (**E**) Percentage force drop following 20% eccentric contraction and (**F**) specific force of TA muscles from *mdx* mice administered rAAV9-µUtrn-FLAG compared to those from vehicle control mice. Maximal isometric force and cross-sectional area of TA muscles are present in Supplementary Figure S11. All values are presented as the mean ± SEM (N = 8 muscles), and statistical significance was set at *P* ≤ 0.05.

We confirmed the induction of µUtrn expression by all constructs via western blot analysis and immunostaining at day 14 following injection (Fig. 6A,D). Protein levels from constructs under CMV, MHCK7, and SPc5-12 promoters did not differ significantly between each other (Fig. 6B). However, the SPc5-12 promoter induced three-fold lower mRNA levels relative to CMV and MHCK7, as determined via RT-qPCR analysis (Fig. 6C). Restoration of DAGC on the sarcolemma was achieved in all groups, although highest α1-syntrophin expression was observed in the rAAV9-CMV-Hco-µUtrn-treated group (Fig. 6D).

We observed the improvement of tetanic force in all treated TA muscles when compared to the muscles of untreated *mdx* mice. All rAAV9-Promoter-µUtrn variants led to a similar force deficit after eccentric contractions (Fig. 6E). The SPc5-12 promoter group exhibited the best specific force relative to those in the CMV and MHCK7 groups, which was also comparable to the force of wild-type controls (Fig. 6F). Thus, muscle-specific promoters MHCK7 and SPc5-12 were as effective as the constitutive CMV promoter. Further, all tested promoters induced *Hco-*µ*Utrn* expression and functional improvement at comparable levels.

## Discussion

Studies utilizing microdystrophin vectors have previously demonstrated that codon optimization can significantly increase the level of transgene protein^22,23^. The current work focused on μUtrn codon optimization in an attempt to enhance gene expression in the skeletal muscles and heart, thereby contributing to functional improvement following rAAV9-mediated gene transfer. We used the codon bias of the most prevalent muscle proteins, namely actin and myosin (see Supplementary Fig. S1 for details), and optimized 63% of the codons in human µUtrn. It should be noted that although the µUtrn cDNAs were optimized for use in humans, codon usage and transfer RNA frequencies are similar across vertebrates^31^, which suggests that cDNAs were appropriate for the mouse model used in our study. The resulting human codon-optimized µUtrn coding sequence was compared to a previously described murine codon-optimized µUtrn sequence^17^ and a control non-optimized human sequence^32^ with the same domain structure. A similar strategy was followed by Song and colleagues^20^ for both human and canine sequences. In addition to codon-optimization, they focused on µUtrn structure and chose internal deletion in order to avoid disruption of the native triple helical repeat within the Rod-domain.

In the present work, codon optimization of human μUtrn resulted in high levels of µUtrn expression in TA muscles following intramuscular administration of 2 × 10^12^ vg rAAV9. Protein expression from Hco-µUtrn exceeded that from H-µUtrn (Fig. 2A,B) and protected muscle against contraction-induced injury (Fig. 2F). Unexpectedly, TA muscles of *mdx* mice, treated with rAAV9-H-µUtrn, exhibited the most pronounced improvement in specific force (Fig. 2G).

Original murine µUtrn was previously tested in *mdx:utrn*^−/−^ mice using an rAAV6 vector for gene delivery^17^. After the systemic administration of rAAV6-µUtrn (3 × 10^12^ vg/mouse) to 4-week-old *mdx:utrn*^−/−^ mice, authors confirmed alleviation of the dystrophic phenotype and increased force production. In similar experimental conditions (1.2 × 10^13^ vg/mice, 6-week-old *mdx* mice), we detected high µUtrn expression levels in striated muscles, heart, diaphragm, and functional improvement at several time points within 20 weeks after the injection of rAAV9-Hco-µUtrn. However, using small animal numbers for functional tests doesn’t allow detection of significant changes throughout the experiment and should be considered as a limitation of present study. Further, no toxicity was observed following systemic vector delivery. Treatment also lowered serum CK level, improved the specific force of isolated TA muscle, and protected against contraction-induced injury (Fig. 2). In a recent study Banks et al.^21^ demonstrated that the number of µUtrn-positive myofibrils depends on the experiment duration. They observed high levels of µUtrn expression two weeks after the intravenous infusion of rAAV6-CK8e-Flag-µUtrn to mdx^4cv^ mice, which was markedly reduced at later time points. Considering that µUtrn co-exists with full-length utrophin at the sarcolemma and the latter is found at higher levels in type 1a, 2a, and 2d fibers^21^, which are less protected by µUtrn, their results suggested that endogenous utrophin expression may adversely impact the therapeutic potential of µUtrn. In the present study, we detected similar mRNA levels in TA muscle after direct intramuscular and intravenous injection of rAAV9-CMV-µUtrn into *mdx* mice expressing full-length utrophin (Figs. 2C and 3B). Both injections protected muscle from force drop during repeated eccentric construction, and short-term analysis revealed more pronounced functional improvement (Fig. 2F–2 weeks and 3g–20 weeks), which indirectly confirms the findings of Banks and colleagues.

Safety evaluation of novel gene therapy vectors in wild-type animals is an essential step in the development of these therapeutics^2^. Herein, we tested FLAG-modified Hco-µUtrn in wild-type rats and did not observe any adverse effects after intravenous injections of 2 × 10^14^ vg kg^−1^ and 6 × 10^14^ vg kg^−1^ at time points corresponding to acute and subchronic toxicity (Fig. 5, Table 1, and Supplementary Fig. S5–S7). *Hco-*µ*Utrn* was successfully delivered and expressed in striated muscle, heart, diaphragm, and the liver (Fig. 5B). Taking into consideration that a high dose of administered rAAV9-Hco-µUtrn harboring the CMV promoter might lead to some transgene expression in non-target tissues, µUtrn gene therapy can be considered extremely safe. However, further studies on the long-term effects of µUtrn expression and safety evaluation in large animals will add to existing evidence in favor of the safety of this therapeutic approach^33–35^.

As previously mentioned, the generation of immune responses against the foreign dystrophin protein represents a major concern in dystrophin replacement therapy. An immune response against microdystrophin following the first intramuscular vector injection was previously confirmed in human patients receiving AAV2.5^3^. Various immune reactions have also been observed in other clinical trials evaluating systemic rAAV-based delivery of microdystrophin^2,36^. In one trial (NCT03368742), two patients treated with high (2 × 10^14^ vg kg^−1^) and low (5 × 10^13^ vg kg^−1^) doses exhibited activation of the complement system with signs of cardiopulmonary decline, although all transient serious adverse events were fully resolved. In another clinical trial (NCT03362502), immune responses measured by T-cell responses on ELISPOT and neutralizing antibody levels were documented in all participants^37^. The great advantage of utrophin is its non-immunogenic nature, achieved through expression in the thymus during the early stages of development^38^. The lower immunogenicity of utrophin was first demonstrated in a study comparing miniutrophin and minidystrophin delivery using an adenoviral vector^39^. In a recent study, Song et al.^20^ compared the immunogenicity of microdystrophin and µUtrn, confirming this notion. In the current work, we also demonstrated the lower immunogenicity of µUtrn constructs over µDys. Further, we investigated the humoral and cellular immune responses against µUtrn and µDys proteins. As expected, M-µUtrn delivery results in lower CD8 + CTL infiltration rates than other transgene products in the muscles of *mdx* mice. Hco-µUtrn-treated muscles also showed lower infiltrating CTL number relative to non-treated *mdx* and µDys groups. The presence of transgene-specific antibodies in the sera of *mdx* mice during long-term monitoring also confirmed the advantage of µUtrn over µDys, although the sample size was low (n = 4), which is a significant limitation of the study.

Ubiquitous promoters, such as CMV, RSV, and EF1a, provide strong and robust transgene expression but may trigger potent immune responses in patients^3^. Thus, current clinical trials of microdystrophin delivery evaluate constructs under muscle-specific promoters CK8^40^, MHCK7^29^ and MCK^41^. Thus, the latest research on AAV-mediated microutrophin delivery has also focused on the usage of muscle-specific promoters^20,21^. In the present study, the CMV promoter facilitated high *Hco-*µ*Utrn* expression in the rat liver, comparable to levels in muscle tissue (Fig. 4B) although without any toxicity after systemic AAV9-CMV-Hco-µUtrn-FLAG delivery. We decided to compare the constitutive CMV promoter with rationally designed muscle-specific natural promoter MHCK7^29^ and synthetic promoter SPc5-12^30^. Following intramuscular injection, muscle-specific promoters demonstrated high activity, comparable to that of the CMV promoter. All analyzed promoters ensured sufficient therapeutic protein expression, resulting in restoration of the DAGC as well as functional recovery. For a more comprehensive comparison, better examination of functional changes and potential toxicity, we plan to extend our data with systemic delivery of muscle-specific promoters-driven µUtrn. Although the lack of these experiments is a limitation of the present study, we want to note that the possible off-target effects are expected to be higher when using a constitutive promoter such as CMV promoter. However, we did not observe any toxicity in histopathology, hematology, blood and urine biochemistry and other parameters after high-dose AAV9-CMV-Hco-µUtrn-FLAG delivery studies on rats (Supplementary Fig. S5–S7).

Taken together our results demonstrated the potential of novel human codon-optimized µUtrn delivered via rAAV9 as gene therapy for DMD. Short-term evaluation of intramuscular delivery as well as long-term studies of systemic administration in *mdx* mice confirmed transgene functionality. Further, extensive toxicity studies in wild-type rats confirmed the safety of our gene therapy. We also demonstrated that muscle-specific promoters MHCK7 and SPc5-12 drove sufficient rAAV9-µUtrn expression for the amelioration of the dystrophic phenotype in *mdx* mice. The current results establish a basis for the further development of human codon-optimized µUtrn combined with muscle-specific promoters as a safe and efficient gene therapy for DMD.

## Materials and methods

### Ethics

All animal procedures were performed in accordance with Directive 2010/63/EU is the European Union (EU) legislation "on the protection of animals used for scientific purposes" and Animal Research: Reporting of In Vivo Experiments (ARRIVE) guidelines and regulations. Experiments were approved by the Local Ethics Committees of Institute of Gene Biology and Belgorod State National Research University.

### µUtrn design and DNA constructs

To design human µUtrn with a ΔR4-R21/ΔCT domain structure, the full-length human utrophin protein sequence (UniProtKB—P46939) was aligned to the murine µUtrn protein (GenBank: ABY20737.1) previously reported by Odom et al.^17^ Protein segments corresponding to 1-685aa, 2690-3165aa, 3431-3433aa in human utrophin were selected, and human µUtrn cDNA (*H-*µ*Utrn*) was created. The nucleotide sequence of H-µUtrn was then codon-optimized (*Hco-*µ*Utrn,* GenBank: OK094718) based on the codon usage in skeletal muscle and cardiac cells (actin, myosin). Details of the codon optimization procedure are provided in the Supplemental Information. All µUtrn cDNAs were modified to include a consensus Kozak sequence. cDNA synthesis was carried out by Shinegene Bio-Technologies, Inc. (Shanghai, China). Transgenes were cloned into a pAAV-MCS vector (Agilent Technologies), and the engineered plasmids were designated as pAAV-CMV-M-µUtrn, pAAV-CMV-H-µUtrn, and pAAV-CMV-Hco-µUtrn, respectively. To generate pAAV-CMV-Hco-µUtrn-FLAG, human codon-optimized µ*Utrn* was fused with a C-terminal FLAG epitope via PCR using the GenPak™ PCR kit (Russia) with the following primers: Hco-µUtrn-FseI-F 5’-tgcgtggacatgtgcctg-3’ and mi-C-Flag-R 5’-ATGgaattcTTACTTGTCGTCATCGTCTTTGTAGTCcatggcctgggtctccaggtt-3’. In pAAV-MHCK7-Hco-µUtrn-FLAG and pAAV-SPc5-12-Hco-µUtrn-FLAG constructs, promoters MHCK7 and SPc5-12 were cloned under MluI/SacI restriction sites, replacing the CMV promoter in pAAV-CMV-Hco-µUtrn-FLAG.

### rAAV9 vector production

rAAV9 encoding µ*Utrn* and µ*Dys* were produced via the triple transfection method as previously described^42^. Briefly, adherent HEK293T (ATCC) cells were maintained in high-glucose DMEM (Gibco, USA) supplemented with 10% v/v fetal bovine serum (FBS, Biosera Europe, France) and Pen-Strep. Transfection was performed using linear PEI (polyethyleneimine) (Polysciences, USA) and the mix of plasmids for virus assembly: (i) pAAV-µUtrn or pAAV-µDys expression vectors, (ii) rep2-cap9-modified AAV plasmid (Penn Vector Core, USA), and (iii) pHelper (Agilent, USA). Seventy-two h after transfection, the cells were lysed with 0,5% v/v Triton X-100, 5 µg mL^−1^ RNAse A, and 10 µg mL^−1^ DNAse A (Calbiochem, USA) for 1 h at 37 °C with shaking. Clarified and concentrated virus suspension was loaded on a modified version of an iodixanol (Sigma Aldrich, USA) density step gradient (60%, 40%, and 25%)^43^ and subjected to ultracentrifugation for 1 h at 350 000 g and 18 °C. The AAV-containing fraction was dialyzed using a 100 kDa membrane against the storage buffer (PBS, 350 mM NaCl, 0.1% Pluronic F-68 (Gibco), and the purified suspension was further concentrated and sterilized^44^. A quantitative PCR-based method was used to determine the encapsidated vector genome (vg) titer using primers and a fluorescence probe targeting ITR sequences^45^. Absence of protein impurities in preparations was confirmed via Silver staining (ThermoFisher, USA) (Supplementary Fig. S9).

### rAAV9 administration and tissue collection in mice

Dystrophin-deficient C57BL/10ScSn-Dmd^mdx^/J (*mdx*) male mice and C57BL/10ScSnJ (B10, parental strain) male mice were obtained from Jackson Laboratory. Before AAV-µUtrn administration, mice were anesthetized with tiletamine hydrochloride/zolazepam hydrochloride mix (50 mg/kg) and xylazine (5 mg/ml) via intraperitoneal injections. For intramuscular delivery (i/m), *mdx* mice received injections of AAV-µUtrn diluted in 40 μl DPBS into each TA muscle (2 × 10^12^ vg/muscle). For intravenous delivery (i/v), *mdx* mice received injections of AAV-µUtrn diluted in 300 µl DPBS into the retroorbital venous sinus (150 µl per each sinus, 6 × 10^14^ vg kg^−1^). Control mice received injections of DPBS in the corresponding volume.

Mice were euthanized at 2 weeks post-injection for i/m delivery or 20 weeks for i/v delivery, after terminal physiological tests. Muscles were collected for protein, RNA, and immunofluorescence analysis. Muscles for protein and RNA extraction were snap-frozen in liquid nitrogen, while tissues for staining were embedded in mounting medium Tissue-Tek OCT Compound (Sakura Finetek, Japan) and frozen in isopentane (PanReac, Spain) precooled with liquid nitrogen. All samples were stored at -70 °C before analysis.

### CK

Blood was collected from jugular veins before euthanasia. Blood was left for 15 min at room temperature and centrifuged (3500 g for 10 min at 4 °C) to promote clot formation and serum collection. CK activity was determined using the Creatine Kinase Activity Assay Kit (Sigma-Aldrich) and the Synergy 4 instrument (BioTek Instruments, USA).

### Histopathology

Organs were weighed and fixed in 10% buffered formalin and embedded in paraffin. 5-µm-thick transverse sections were stained with H&E according to routine procedure described in Slaoui et al.^46^. Images of H&E-stained sections were acquired with a Zeiss Axiocam camera at 200 × magnification.

### Toxicity assessment

Toxicity studies were conducted at Belgorod State University (Russia). The study included 30 male Wistar rats (21 days old). rAAV9-Hco-µUtrn-FLAG was tested at two doses: 2 × 10^14^ vg kg^−1^ and 6 × 10^14^ vg kg^−1^ after single injection via intravenous route (lateral tail vein). Euthanasia was performed by exsanguination with cardiac puncture under isoflurane inhalation. Animal behavior in the open field test was recorded daily using an ActiTrack system (Panlab, Spain). A modified Irwin test was performed daily for general assessment of the animals welfare. Terminal procedures, consisting of electrocardiography (ECG), blood pressure measurement, ophthalmoscopy, collection of whole blood, serum, and urine samples as well as necropsy were performed on the days 3 (½ animals) and 14 (½ animals). Collected urine was analyzed using test strips and a semi-automatic urine analyzer Urit 180 Vet (URIT Medical Electronic Group, China). Complete blood count analysis was performed with use of automatic hematology analyzer CELL-DYN 3700 (Abbott Diagnostics, USA). Biochemical blood analysis was performed on an A25 analyzer (Biosystems, Spain) using reagents and control materials from Hospitex Diagnostics. ECG was performed using the ECG-1003 DIXION machine (Dixon Technologies, India).

### Immunofluorescence

Transverse 10-μm-thick sections of muscles were obtained using a Leica CM 1510-1 cryostat. Muscle sections were fixed with 4% paraformaldehyde (AppliChem, Germany) and 2% D( +)Sucrose (AppliChem) in PBS for 30 min at room temperature and permeabilized in PBST (0.01% Triton X-100 solution in PBS). Non-specific antibody binding was blocked with 3% bovine serum albumin (BSA, PanEco, Russia) solution in PBST for an hour at room temperature. Sections were stained with rabbit polyclonal primary antibodies against dystrophin (C357462, 1:200, LS-Bio, USA), utrophin (CAU22354, 1:800, Biomatik, Canada), other DAGC components (ab188873, 1:500, ab189254, 1:1000, Abcam, UK), Flag-epitope (F 7425, 1:200, Sigma, Germany), and Alexa Fluor-labeled secondary antibodies (ab150077, 1:1000, Abcam; A21072, 1:1000, Invitrogen). CD8 + cells were stained with directly-labeled antibodies (42-0081-82, 1:40, ThermoFisher). Nuclei were counterstained with Hoechst 33,342 (ThermoFisher) or DAPI (ThermoFisher). Antibodies and nuclear dyes were diluted in blocking buffer. Incubation with primary antibodies was carried out overnight at 4C, followed by washes in PBST and incubation with secondary antibodies for 1 h. Muscle cryosections were mounted in ProLong Gold antifade medium (Invitrogen, USA). Appropriate negative tissue controls and isotype controls were implemented in the experiments. Fluorescence images were captured on Zeiss LSM 880 and Leica Stellaris 5 confocal microscopes.

### In vivo physiological testing in *mdx* mice

The wire hanging test was performed for each mouse every week according to the protocol described by Aartsma-Rus and van Putten^47^. A 55-cm-wide 2-mm-thick metallic wire was installed 37 cm above a layer of bedding. Each mouse was given three trials to hang on the wire by the forelegs with a 30-s recovery period between trials. The maximum hanging time of the three trials was recorded and used as an outcome measure.

Measurement of isometric muscle force was performed for each mouse at the end of the experiment according to previously described protocols^48–50^. Briefly, the TA muscle was stimulated with 0.2 ms pulses via two stainless steel electrodes that penetrated the skin on either side of the peroneal nerve near the knee. The muscle was adjusted to an optimum length (*L*o) to produce the maximum tetanic force (*P*o). *L*o and TA mass were recorded and used for normalization to the physiological cross-sectional area (CSA = [*L*o × density]/mass) and the calculation of the specific tetanic force (*sPo*).

Susceptibility to eccentric contraction-induced injury was measured during a series of seven eccentric contractions. TA muscle was stimulated (100 Hz) for 500 ms at Lo to achieve Po and then stretched at 1 mm/s until it was 20% longer than its *L*o, held at this length for 500 ms, and then returned to its original length at the same rate. The deficit of force at each lengthening contraction (P1-P7, P1 = Po) was calculated as (Po-P2-7)/Po × 100%.

### Western blotting

100 mg of muscle tissue was grinded in liquid nitrogen, resuspended in 1 ml of lysis buffer (110 mM Tris–HCl pH 7,8, 150 mM NaCl, 3% SDS, 1 mM EDTA, 10% glycerol, Bromophenol Blue 0.01% Protease inhibitor cocktail (Roche, Switzerland)) and incubated at 95 °C for 5 min. 10–20 μg of tissue lysate was loaded per lane of 8% SDS–polyacrylamide gel. Proteins were wet transferred to a PVDF membrane in Mini Trans-Blot Cell (Bio-Rad, USA). Membrane was blocked in 5% dry milk in TBS-T overnight. µUtrn and µDys were detected using a rabbit polyclonal antibody (CAU22354, 1:500, Biomatic) and mouse monoclonal antibodies (NCL-DYSB, 1:500, Leica, Germany), respectively. Actin and GAPDH were used as loading controls (A2103, 1:10,000, Sigma-Aldrich; ABS16, 1:5000, Sigma-Aldrich). Corresponding species-specific antibodies conjugated with horseradish peroxidase were used as secondary antibodies (1,706,515, 1,706,516, 1:3000, Bio-Rad). Incubation with primary antibodies was carried out for 2 h at room temperature, followed by washes in TBS-T and incubation with secondary antibodies for 1 h. For humoral immunity assessment, lysates of HEK293T cells transfected with the appropriate transgene were loaded onto a gel. Membrane was blocked in 5% dry milk in TBS-T for 1 h followed by overnight incubation with murine serum samples (1:100) at 4 °C. Protein detection and quantification were performed using the iBright FL1500 Scanner (ThermoFisher) and iBright analysis software.

### RT-qPCR analysis

Total RNA was isolated using the ReliaPrep RNA Tissue Miniprep System (Promega, USA) according to the manufacturer's protocol. Purified RNA was treated with RNase-free DNase I (BioLabs, USA) and reverse transcribed using the MMLV RT kit (Evrogen, Russia). Amplification was performed on a Real-Time CFX 96 Touch amplifier (Bio-Rad) using SybrGreen master mix (Evrogen) and primers specific to the transgenes: 5’-TTCAACTACGACGTGTGC-3’ and 5’-TCACATGGCCTGGGTCTC-3’ for *M-*µ*Utrn*, 5’-GACTAGAAGATTCCTCCAACCA-3’ and 5’-TCTGAGTTTCTCCAAATCCAC-3’ for *H-*µ*Utrn*, 5’-TTCAACTACGACGTGTGC-3’ and 5’-TCACATGGCCTGGGTCTC-3’ for *Hco-*µ*Utrn*. *Rpl13a*, *Ap3d1*, *Csnk2a2* (mouse) and *Ankrd27*, *Hprt1* (rat) were analyzed as housekeeping genes for the normalization of gene expression^51,52^. The following qPCR conditions were used: 94 °C–15 s, 55/61 °C–15 s, 72 °C–15 s (+ fluorescence measurement). The copy number of utrophin transcripts per 1 µg of total cDNA was calculated based on a standard curve built with serial dilutions of the reference plasmid containing the corresponding CDS (*M-*µ*Utrn*, *H-*µ*Utrn*, and *Hco-*µ*Utrn*).

### Immunofluorescence analysis

Quantitative analysis of immunofluorescence images was performed using the CellProfiler 4.2.1 software in order to compare the degree of DAGC recovery and the numbers of infiltrating CD8 + cells after treatment^53^. The order of selected modules can be found in Supplementary Files S3-S4, and the created pipelines will be available for download at www.cellprofiler.org.

### Statistical analysis

Statistical analysis was performed using GraphPad Prism 9 software. Differences between groups were analyzed using the Kruskell-Wallis test, corrected for Dunn’s multiple comparisons. Differences between the control *mdx* group and other groups with *P* < 0.05 were considered statistically significant and marked *. Data in the text, tables, and graphs are presented as the mean ± standard deviation or standard error of the mean (SEM), as stated in the figure legends. The number of measurements is shown in brackets.

## Supplementary Information

Supplementary Information.

## References

[1] Mendell JR, Lloyd-Puryear M (2013). Report of MDA muscle disease symposium on newborn screening for Duchenne muscular dystrophy. Muscle Nerve.

[2] Duan D (2018). Systemic AAV micro-dystrophin gene therapy for duchenne muscular dystrophy. Mol. Ther..

[3] Mendell JR (2010). Dystrophin immunity in Duchenne’s muscular dystrophy. N. Engl. J. Med..

[4] Gramolini AO, Jasmin BJ (1998). Molecular mechanisms and putative signalling events controlling utrophin expression in mammalian skeletal muscle fibres. Neuromuscul. Disord. NMD.

[5] Clerk A, Morris GE, Dubowitz V, Davies KE, Sewry CA (1993). Dystrophin-related protein, utrophin, in normal and dystrophic human fetal skeletal muscle. Histochem. J..

[6] Tinsley J (1998). Expression of full-length utrophin prevents muscular dystrophy in mdx mice. Nat. Med..

[7] Fisher R (2001). Non-toxic ubiquitous over-expression of utrophin in the mdx mouse. Neuromuscul. Disord. NMD.

[8] Krag TOB (2004). Heregulin ameliorates the dystrophic phenotype in mdx mice. Proc. Natl. Acad. Sci. U. S. A..

[9] Moorwood C (2011). Drug discovery for Duchenne muscular dystrophy via utrophin promoter activation screening. PloS One.

[10] Tinsley JM (2011). Daily treatment with SMTC1100, a novel small molecule utrophin upregulator, dramatically reduces the dystrophic symptoms in the mdx mouse. PloS One.

[11] Mattei E (2007). Utrophin up-regulation by an artificial transcription factor in transgenic mice. PloS One.

[12] Pisani C (2018). Utrophin up-regulation by artificial transcription factors induces muscle rescue and impacts the neuromuscular junction in mdx mice. Biochim. Biophys. Acta Mol. Basis Dis..

[13] Amenta AR (2011). Biglycan recruits utrophin to the sarcolemma and counters dystrophic pathology in mdx mice. Proc. Natl. Acad. Sci. U. S. A..

[14] Ito M, Ehara Y, Li J, Inada K, Ohno K (2017). Protein-anchoring therapy of biglycan for mdx mouse model of Duchenne muscular dystrophy. Hum. Gene Ther..

[15] Cerletti M (2003). Dystrophic phenotype of canine X-linked muscular dystrophy is mitigated by adenovirus-mediated utrophin gene transfer. Gene Ther..

[16] Harper SQ (2002). Modular flexibility of dystrophin: implications for gene therapy of Duchenne muscular dystrophy. Nat. Med..

[17] Odom GL, Gregorevic P, Allen JM, Finn E, Chamberlain JS (2008). Microutrophin delivery through rAAV6 increases lifespan and improves muscle function in dystrophic Dystrophin/Utrophin-deficient mice. Mol. Ther. J. Am. Soc. Gene Ther..

[18] Sonnemann KJ (2009). Functional substitution by TAT-utrophin in dystrophin-deficient mice. PLoS Med..

[19] Kennedy TL (2018). Micro-utrophin improves cardiac and skeletal muscle function of severely affected D2/mdx mice. Mol. Ther. Methods Clin. Dev..

[20] Song Y (2019). Non-immunogenic utrophin gene therapy for the treatment of muscular dystrophy animal models. Nat. Med..

[21] Banks GB, Chamberlain JS, Odom GL (2020). Microutrophin expression in dystrophic mice displays myofiber type differences in therapeutic effects. PLoS Genet..

[22] Foster H (2008). Codon and mRNA sequence optimization of microdystrophin transgenes improves expression and physiological outcome in dystrophic mdx mice following AAV2/8 gene transfer. Mol. Ther. J. Am. Soc. Gene Ther..

[23] Athanasopoulos T, Foster H, Foster K, Dickson G (2011). Codon optimization of the microdystrophin gene for Duchene muscular dystrophy gene therapy. Methods Mol. Biol. Clifton NJ.

[24] Skopenkova VV, Egorova TV, Bardina MV (2021). Muscle-specific promoters for gene therapy. Acta Naturae.

[25] Spencer MJ, Montecino-Rodriguez E, Dorshkind K, Tidball JG (2001). Helper (CD4(+)) and cytotoxic (CD8(+)) T cells promote the pathology of dystrophin-deficient muscle. Clin. Immunol. Orlando Fla.

[26] Flotte TR (2011). Phase 2 clinical trial of a recombinant adeno-associated viral vector expressing α1-antitrypsin: interim results. Hum. Gene Ther..

[27] Gernoux G (2020). Muscle-directed delivery of an AAV1 vector leads to capsid-specific T cell exhaustion in nonhuman primates and humans. Mol. Ther. J. Am. Soc. Gene Ther..

[28] Ferla R (2017). Non-clinical safety and efficacy of an AAV2/8 vector administered intravenously for treatment of mucopolysaccharidosis type VI. Mol. Ther. Methods Clin. Dev..

[29] Salva MZ (2007). Design of tissue-specific regulatory cassettes for high-level rAAV-mediated expression in skeletal and cardiac muscle. Mol. Ther. J. Am. Soc. Gene Ther..

[30] Li X, Eastman EM, Schwartz RJ, Draghia-Akli R (1999). Synthetic muscle promoters: activities exceeding naturally occurring regulatory sequences. Nat. Biotechnol..

[31] Hastings KE, Emerson CP (1983). Codon usage in muscle genes and liver genes. J. Mol. Evol..

[32] Stedman, H. H., Su, L. T. & Mitchell, M. A. Microutrophin and uses thereof. (2010).

[33] Duan D (2015). Duchenne muscular dystrophy gene therapy in the canine model. Hum. Gene Ther. Clin. Dev..

[34] Hinderer C (2018). Severe toxicity in nonhuman primates and piglets following high-dose intravenous administration of an adeno-associated virus vector expressing human SMN. Hum. Gene Ther..

[35] Rodino-Klapac LR (2010). Persistent expression of FLAG-tagged micro dystrophin in nonhuman primates following intramuscular and vascular delivery. Mol. Ther..

[36] Ronzitti G, Gross D-A, Mingozzi F (2020). Human immune responses to adeno-associated virus (AAV) vectors. Front. Immunol..

[37] Pfizer Presents Initial Clinical Data on Phase 1b Gene Therapy Study for Duchenne Muscular Dystrophy (DMD) |Pfizer. https://www.pfizer.com/news/press-release/press-release-detail/pfizer_presents_initial_clinical_data_on_phase_1b_gene_therapy_study_for_duchenne_muscular_dystrophy_dmd.

[38] Mesnard-Rouiller L, Bismuth J, Wakkach A, Poëa-Guyon S, Berrih-Aknin S (2004). Thymic myoid cells express high levels of muscle genes. J. Neuroimmunol..

[39] Ebihara S (2000). Differential effects of dystrophin and utrophin gene transfer in immunocompetent muscular dystrophy (mdx) mice. Physiol. Genomics.

[40] Hakim CH (2017). A five-repeat micro-dystrophin gene ameliorated dystrophic phenotype in the severe DBA/2J-mdx model of duchenne muscular dystrophy. Mol. Ther. Methods Clin. Dev..

[41] Wang B (2008). Construction and analysis of compact muscle-specific promoters for AAV vectors. Gene Ther..

[42] Danilov KA (2020). In vitro assay for the efficacy assessment of AAV vectors expressing microdystrophin. Exp. Cell Res..

[43] Buclez P-O (2016). Rapid, scalable, and low-cost purification of recombinant adeno-associated virus produced by baculovirus expression vector system. Mol. Ther. Methods Clin. Dev..

[44] Zolotukhin S (1999). Recombinant adeno-associated virus purification using novel methods improves infectious titer and yield. Gene Ther..

[45] Aurnhammer C (2012). Universal real-time PCR for the detection and quantification of adeno-associated virus serotype 2-derived inverted terminal repeat sequences. Hum. Gene Ther. Methods.

[46] Slaoui M, Bauchet A-L, Fiette L (2017). Tissue sampling and processing for histopathology evaluation. Methods Mol. Biol. Clifton NJ.

[47] Aartsma-Rus A, van Putten M (2014). Assessing functional performance in the mdx mouse model. J. Vis. Exp. JoVE.

[48] Egorova TV (2019). CRISPR/Cas9-generated mouse model of Duchenne muscular dystrophy recapitulating a newly identified large 430 kb deletion in the human DMD gene. Dis. Model. Mech..

[49] Chan S, Head SI, Morley JW (2007). Branched fibers in dystrophic mdx muscle are associated with a loss of force following lengthening contractions. Am. J. Physiol. Cell Physiol..

[50] Dellorusso C, Crawford RW, Chamberlain JS, Brooks SV (2001). Tibialis anterior muscles in mdx mice are highly susceptible to contraction-induced injury. J. Muscle Res. Cell Motil..

[51] Wang X (2019). Identification of suitable reference genes for gene expression studies in rat skeletal muscle following sciatic nerve crush injury. Mol. Med. Rep..

[52] Hildyard JCW, Finch AM, Wells DJ (2019). Identification of qPCR reference genes suitable for normalizing gene expression in the mdx mouse model of Duchenne muscular dystrophy. PLOS ONE.

[53] Jones TR (2008). Cell profiler analyst: data exploration and analysis software for complex image-based screens. BMC Bioinf..

