# Supplementary material for: Therapeutic potential of highly functional codon-optimized microutrophin for muscle-specific expression
Source: Sci Rep. 2022 Jan 17;12:848. doi: 10.1038/s41598-022-04892-x (PMC8764061; doi:10.1038/s41598-022-04892-x)
Supplement: Supplementary file 1 — Supplementary Information. [file 41598_2022_4892_MOESM1_ESM.pdf]

Supplementary information

Therapeutic potential of highly functional codon-optimized microutrophin for muscle-specific expression

Anna V. Starikova[1+], Victoria V. Skopenkova[1, 2+], Anna V. Polikarpova[1], Denis A. Reshetov[3], Svetlana G. Vassilieva[1], Oleg A. Velyaev[1], Anna A. Shmidt[1, 2], Irina M. Savchenko[1], Vladislav O. Soldatov[4, 5], Tatiana V. Egorova[1, 6*], Maryana V. Bardina[1, 2, 6]

[1] Institute of Gene Biology, Laboratory of modeling and gene therapy of hereditary diseases, Russian Academy of Sciences, Moscow, 119334 Russia
[2] Institute of Gene Biology, Center for Precision Genome Editing and Genetic Technologies for Biomedicine, Russian Academy of Sciences, Moscow, 119334 Russia
[3] Research centre for Genetic Medicine, Moscow, 117292, Russia
[4] Belgorod State National Research University, Department of Pharmacology and Clinical Pharmacology, Belgorod, 308007 Russia
[5] Institute of Gene Biology, Russian Academy of Sciences, Core Facility Centre, Moscow, 119334 Russia
[6] Marlin Biotech LLC, Sochi, 354340 Russia
[+] These authors contributed equally to this work

* Correspondence should be addressed to T.V.E. (egorovatv@genebiology.ru)

Short title: Codon-optimized microutrophin for DMD therapy

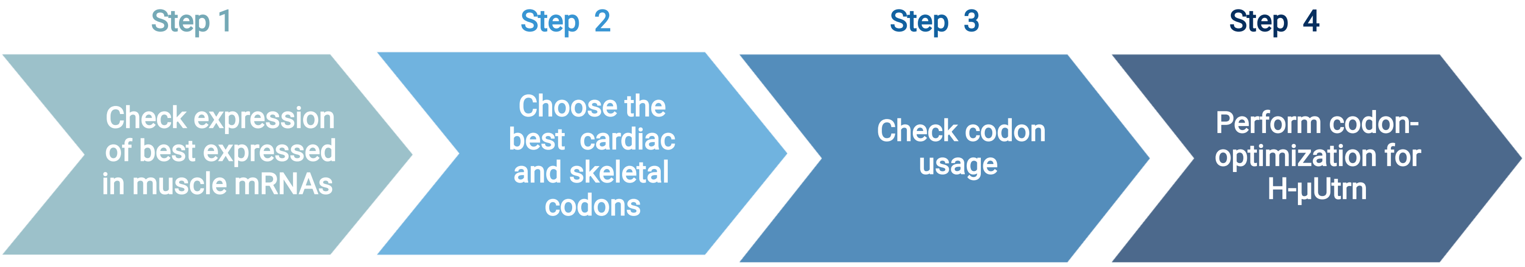

Gene	Heart	Skeletal
MYH1	10	90
MYH2	10	90
MYH3	10	10
MYH4	1	10
MYH6	120	12
MYH7	150	150
MYH7B	10	10
MYH8	1	10
MYH9	100	100
MYH10	12	10
MYH11	12	12
MYH13	1	10
MYH14	12	12
MYH15	5	5
MYH16	10	5
ACTA1	150	150
ACTC1	150	15

Amino acid	Codon
A	GCC
C	TGC
D	GAC
E	GAG
F	TTC
G	GGC
H	CAC
I	ATC
K	AAG
L	CTG
M	ATG
N	AAC
P	CCC
Q	CAG
R	CGC
S	TCC
T	ACC
V	GTG
W	TGG
Y	TAC

Gene	% of optimum codons
ACTA1	78%
MYH9	72%
MYH6	71%
MYH7	69%
MYH14	64%
MYH11	63%
ACTC1	58%
MYH3	54%
MYH2	51%
MYH1	50%
MYH10	46%

Gene	% of optimum codons
**H-µUtrn**	**37%**
**M-µUtrn**	**51%**
**Hco-µUtrn**	**100%**

Supplementary Figure S1. Codon optimization process. Step 1. Obtained the filtered list of the best expressed in muscle mRNAs with threshold "10" (https://www.genecards.org/, RNA seq, Illumina BodyMap; $(100*FPKM)^{-2}$. Step 2. Selected the best cardiac and skeletal codons. Step 3. Calculated optimum codons in actin and myosin. Step 4. Calculated optimum codons in transgenes mRNAs.

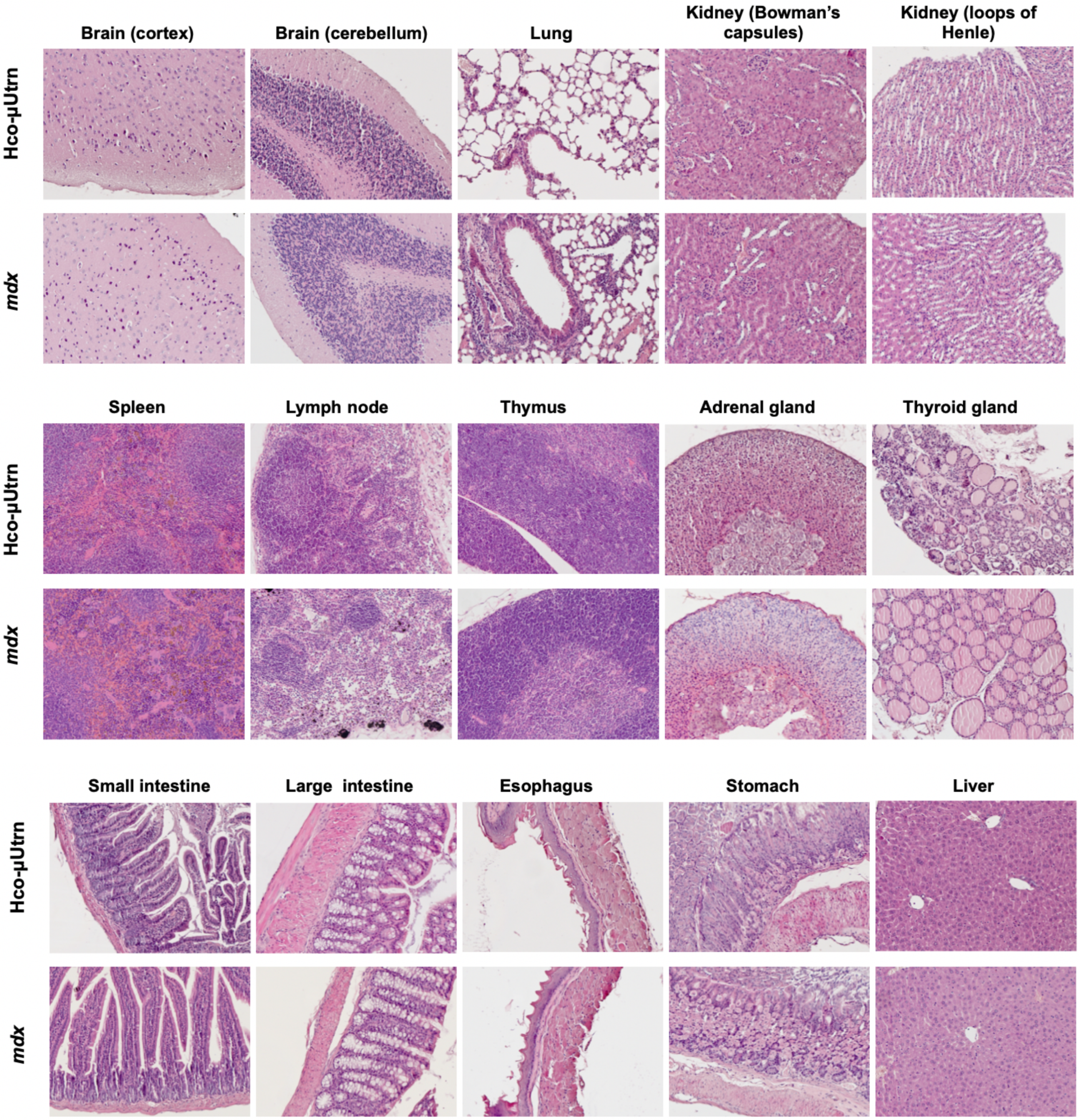

Supplementary Figure S2. Representative images of H&E stained organs collected from mdx mice injected with rAAV9-Hco-µUtrn. High dose (6x10^{14} GC kg^{-1}) rAAV9-Hco-µUtrn delivery in adult *mdx* mice did not alter histology of major internal organs. H&E staining of thyroid gland showed histopathologic findings 20 weeks after injection: reduction in follicles number and hypertrophy of follicles epithelium.

Supplementary File S3. Modules order for “Intensity measurement on sarcolemma” CellProfiler pipeline.

[1] [Images]

[2] [Metadata]

[3] [NamesAndTypes]

[4] [Groups]

[5] [MeasureImageIntensity]

[6] [ImageMath]

[7] [EnhanceOrSuppressFeatures]

[8] [RescaleIntensity]

[9] [Threshold]

[10] [MaskImage]

[11] [MeasureImageIntensity]

[12] [ImageMath]

[13] [MaskImage]

[14] [MeasureImageIntensity]

[15] [MeasureImageIntensity]

[16] [ImageMath]

[17] [EnhanceOrSuppressFeatures]

[18] [RescaleIntensity]

[19] [Threshold]

[20] [MaskImage]

[21] [MeasureImageIntensity]

[22] [ImageMath]

[23] [MaskImage]

[24] [MeasureImageIntensity]

[25] [ExportToSpreadsheet]

Supplementary File S4. Modules order for “CTL counting” CellProfiler pipeline.

[1] [Images]

[2] [Metadata]

[3] [NamesAndTypes]

[4] [Groups]

[5] [ColorToGray]

[6] [CorrectIlluminationCalculate]

[7] [CorrectIlluminationApply]

[8] [GaussianFilter]

[9] [MeasureImageIntensity]

[10] [IdentifyPrimaryObjects]

[11] [CorrectIlluminationCalculate]

[12] [CorrectIlluminationApply]

[13] [GaussianFilter]

[14] [MeasureImageIntensity]

[15] [Threshold]

[16] [IdentifyPrimaryObjects]

[17] [RelateObjects]

[18] [FilterObjects]

[19] [ExportToSpreadsheet]

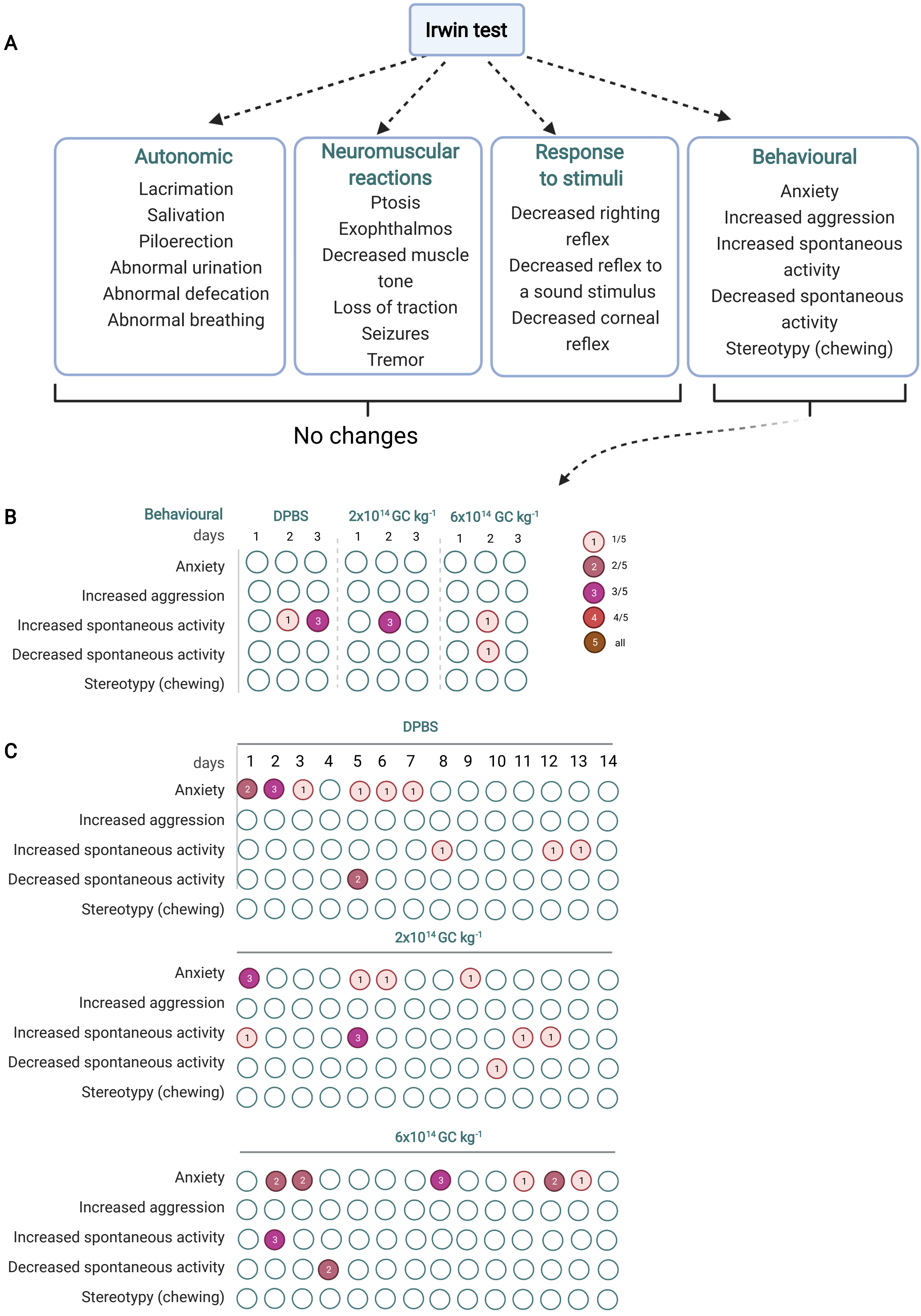

Supplementary Figure S5. Toxicity study in rats. A. Results of daily monitoring according to the Irwin test. B. Groups with 3-day observation. C. Groups with 14-day observation.

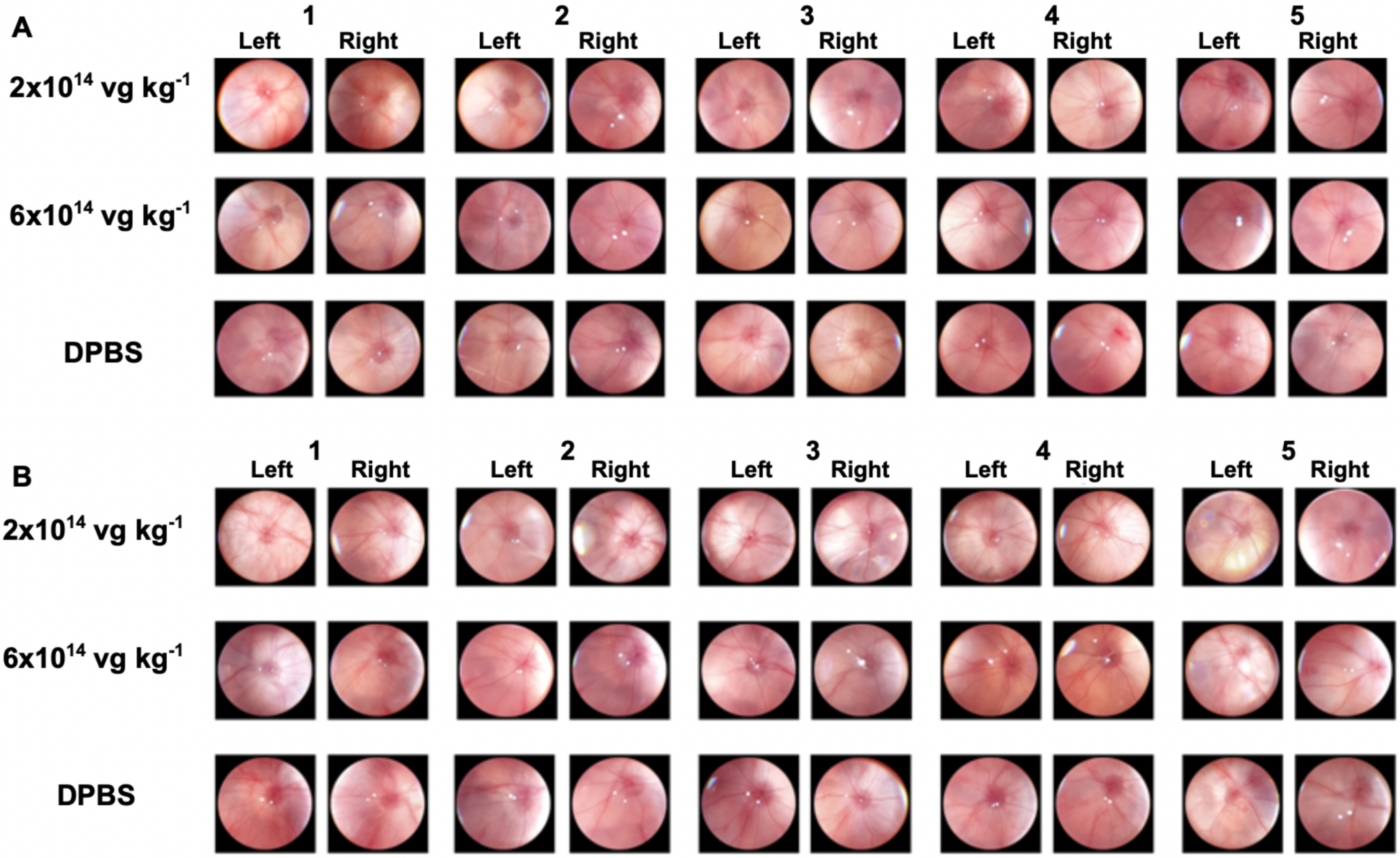

Supplementary Figure S6. A. Ophthalmologic examination of fundus on day 3. B. Ophthalmologic examination of fundus on day 14.

Supplementary Table S7. Toxicity in rats. Clinical urine tests

Day, group	Day 3			Day 14		
	DPBS	$2x10^{14}$ vg kg^{-1}	$6x10^{14}$ vg kg^{-1}	DPBS	$2x10^{14}$ vg kg^{-1}	$6x10^{14}$ vg kg^{-1}
Diuresis (4 hours), µl	270 [52.5; 475]	600 [310; 630]	250 [85; 542.5]	140 [90; 360]	240 [50; 240]	350 [80; 410]
Relative density	1010 [1002.5; 1017.5]	1030 [1020; 1030]	1010 [1002.5; 1020]	1020 [1010; 1020]	1020 [1010; 1030]	1020 [1010; 1030]
pH	6.5 [6.12; 7]	6.5 [6.12; 6.88]	6.5 [6.12; 7]	6.5 [6; 7]	6 [6; 7]	6 [6; 6.5]
Proteinuria (% of animals in the group)	0	0	0	0	0	0
Glucosuria (% of animals in the group)	0	0	0	0	0	0
Bilirubinuria (% of animals in the group)	0	0	0	0	0	0
Leukocyturia (% of animals in the group)	0	0	0	0	0	0
Nitrites (% of animals in the group)	0	0	0	0	0	0
Ketones (% of animals in the group)	0	0	0	0	0	0

Note: Quantitive data are presented as Me [Q1; Q3]. Qualitative data are presented as % of animals in the group displaying the clinical sign.

Supplementary Table S8. rAAV-μUtrn production and purification efficiency

**Virus preparation**	**Number of experiments (n)**	**Virus yield in crude lysate after triple transfection (vg/cm2)***	**Virus yield after purification procedures (vg/cm2)**
AAV9-CMV-μDys	2	$7{,}1\times10^{10} \pm 7{,}8\times10^{9}$	$2{,}2\times10^{10} \pm 1{,}6\times10^{7}$
AAV9-CMV-H-μUtrn	1	$2{,}1\times10^{11}$	5×10^{10}
AAV9-CMV-Hco-μUtrn	5	$6\times10^{10} \pm 2\times10^{10}$	$1{,}6\times10^{10} \pm 7{,}5\times10^{9}$
AAV9-CMV-M-μUtrn	1	$2{,}3\times10^{11}$	$5{,}1\times10^{10}$
AAV9-CMV-Hco-μUtrn	1	$3{,}2\times10^{10}$	$8{,}1\times10^{9}$
AAV9-spc512-Hco-μUtrn-FLAG	1	$7{,}6\times10^{10}$	$1{,}7\times10^{10}$
AAV9-MHCK7-Hco-μUtrn-FLAG	1	$1{,}5\times10^{11}$	$2{,}2\times10^{10}$

** At all steps of the rAAV manufacturing process virus titer was measured by qPCR as described in the Materials and Methods. To compare different virus preparations, values were normalized to the total surface area of cell culture dishes used for each triple transfection and presented as DNase-resistant viral genomes (vg) per cm2.*

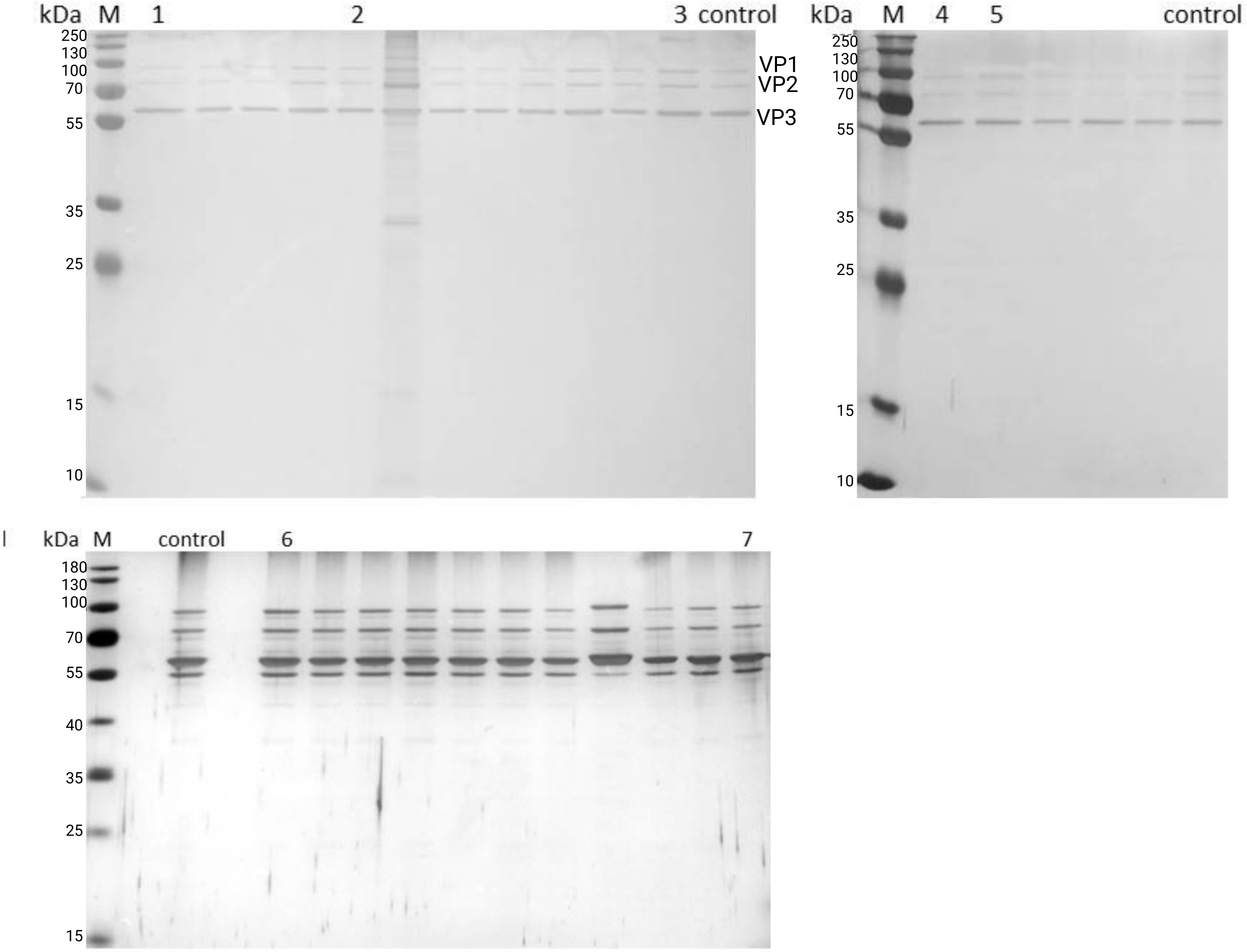

Supplementary Figure S9. Silver-stained gel image of purified AAV vectors with structural proteins VP1, VP2 and VP3.

1 - AAV9-CMV-Hco-µUtrn-FLAG

2 - AAV9-CMV-µDys

3 - AAV9-CMV-Hco-µUtrn

4 - AAV9-MHCK7-Hco-µUtrn-FLAG

5 - AAV9-SPc5-12-Hco-µUtrn-FLAG

6 - AAV9-CMV-M-µUtrn

7 - AAV9-CMV-H-µUtrn

M - PageRuler Protein Ladder.

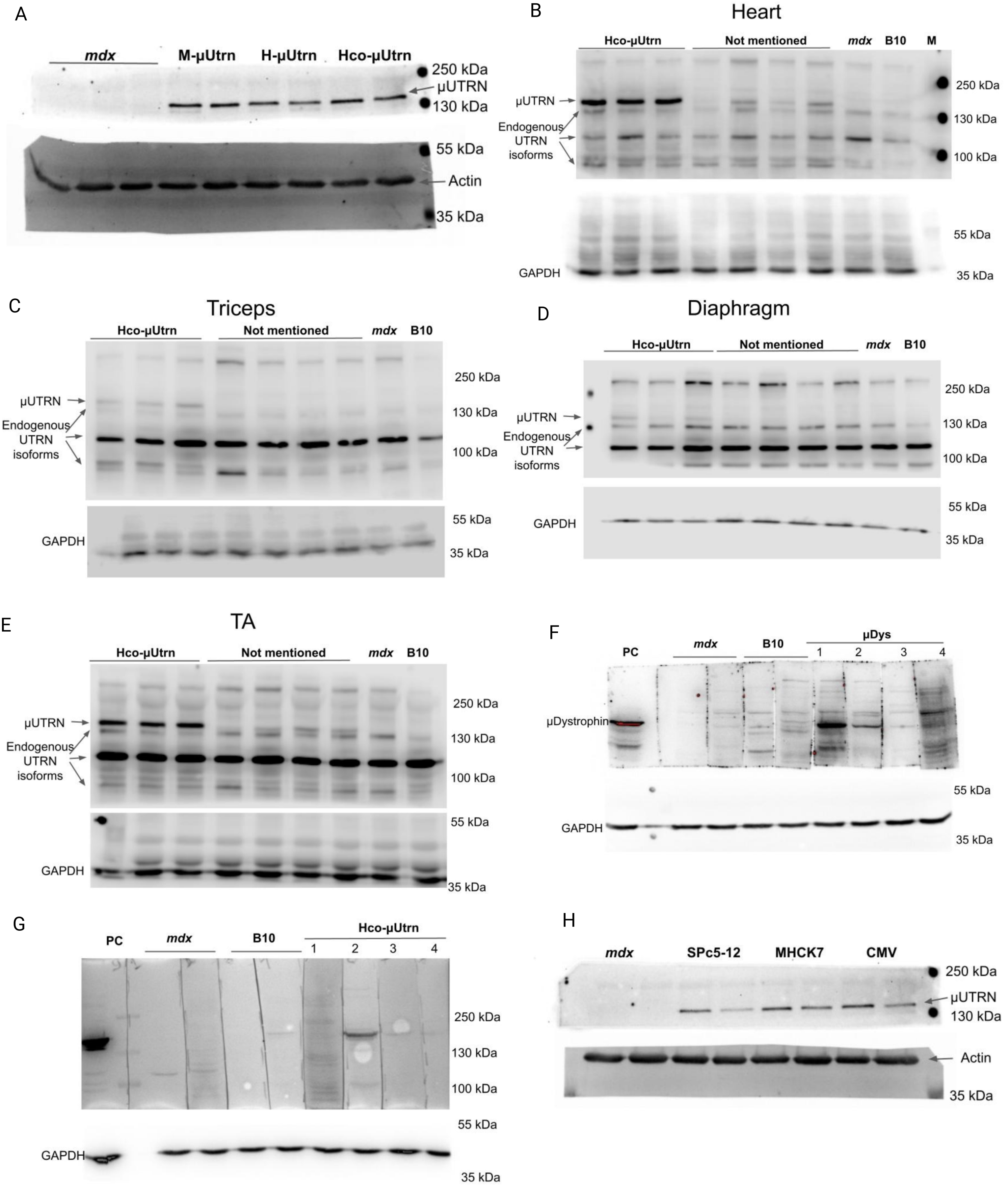

Supplementary Figure S10. Unprocessed western blots . A - Figure 2a. B, C, D, E - Figure 3a. F, G - Figure 3h. H - Figure 6a.

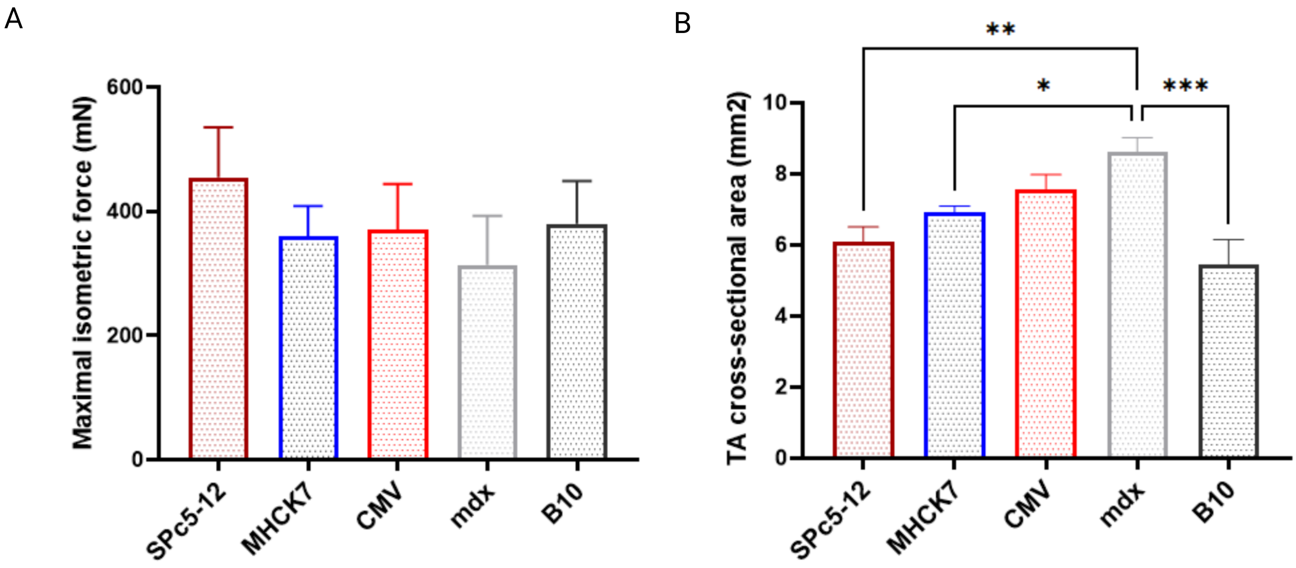

Supplementary Figure S11. A. Maximal isometric force, (B) cross-sectional area of TA muscles from *mdx* mice administered rAAV9-µUtrn-FLAG compared to those from vehicle control mice.
